# FOXP in Tetrapoda: Intrinsically Disordered Regions, Short Linear Motifs and their evolutionary significance

**DOI:** 10.1590/1678-4685-GMB-2016-0115

**Published:** 2017-03-02

**Authors:** Lucas Henriques Viscardi, Luciana Tovo-Rodrigues, Pamela Paré, Nelson Jurandi Rosa Fagundes, Francisco Mauro Salzano, Vanessa Rodrigues Paixão-Côrtes, Claiton Henrique Dotto Bau, Maria Cátira Bortolini

**Affiliations:** 1 Programa de Pós-Graduação em Genética e Biologia Molecular, Departamento de Genética, Universidade Federal do Rio Grande do Sul, Porto Alegre, RS, Brazil.; 2 Programa de Pós-Graduação em Epidemiologia, Universidade Federal de Pelotas, Pelotas, RS, Brazil.; 3 Programa de Pós-Graduação em Genética e Biodiversidade, Instituto de Biologia, Universidade Federal da Bahia, Salvador, BA, Brazil.

**Keywords:** FOXP2, Intrinsically disordered protein regions, forkhead superfamily, short linear motif, molecular evolution

## Abstract

The *FOXP* subfamily is probably the most extensively characterized subfamily of the forkhead superfamily, playing important roles in development and homeostasis in vertebrates. Intrinsically disorder protein regions (IDRs) are protein segments that exhibit multiple physical interactions and play critical roles in various biological processes, including regulation and signaling. IDRs in proteins may play an important role in the evolvability of genetic systems. In this study, we analyzed 77 orthologous *FOXP* genes/proteins from Tetrapoda, regarding protein disorder content and evolutionary rate. We also predicted the number and type of short linear motifs (SLIMs) in the IDRs. Similar levels of protein disorder (approximately 70%) were found for FOXP1, FOXP2, and FOXP4. However, for FOXP3, which is shorter in length and has a more specific function, the disordered content was lower (30%). Mammals showed higher protein disorders for FOXP1 and FOXP4 than non-mammals. Specific analyses related to linear motifs in the four genes showed also a clear differentiation between FOXPs in mammals and non-mammals. We predicted for the first time the role of IDRs and SLIMs in the *FOXP* gene family associated with possible adaptive novelties within Tetrapoda. For instance, we found gain and loss of important phosphorylation sites in the *Homo sapiens* FOXP2 IDR regions, with possible implication for the evolution of human speech.

## Introduction

Members of the *Forkhead box* (*FOX*) gene superfamily have been widely associated with organismal development and are identified by their evolutionary conserved forkhead DNA-binding domain (Lam *et al*., 2013; Morris and Fanucchi, 2016). The *FOXP* subfamily is probably the most extensively characterized subfamily of the forkhead superfamily. The four *FOXP* genes (*FOXP1, FOXP2 FOXP3*, and *FOXP4*) emerged by duplication events during the origin of vertebrates (Santos *et al*., 2011; Song *et al*., 2016). Since the duplication events, paralogues *FOXP1*, *FOXP2*, and *FOXP4* have played an important role in brain, lung, heart, and jaw development in vertebrates, while *FOXP3* has been associated with the development and homeostasis of the immune system, since it is described as a master-regulator of CD4^+^ and CD25^+^ T-cells (Coffer and Burgering, 2004; Akbar *et al*., 2007; Takahashi *et al*., 2008; Benayoun *et al*., 2011; Andersen *et al*., 2012; Lam *et al*., 2013; Cesario *et al*., 2016).

Undoubtedly, the most widely known member of the *FOXP* subfamily is *FOXP2*, as it has attracted the attention of the scientific community and the general media because of its role in the evolution of speech and vocalization in mammals (Zhang *et al*., 2002; Li *et al*., 2007), especially because mutations in this gene promote severe impairment of articulation and grammar in humans (Enard *et al*., 2002; Schön *et al*., 2006; Enard, 2011; Bowers and Konopka, 2012). *FOXP2* is expressed primarily in the brain, where it plays an important role in synapse formation and cell adhesion, as well as in the specification and differentiation of the lung epithelium and gastrointestinal and cardiovascular tissues (Song *et al*., 2013).

Evolutionary studies have been successively improved by incorporating new methodological approaches. Analysis of intrinsically disordered regions (IDRs), which is routinely used in medical and structural biology studies, can also be applied in evolutionary studies because of the possible role of IDRs in the evolvability (evolutionary capacity; Pigliucci, 2008; Xue *et al*., 2010b, 2013) of genetic systems (Neduva and Russell, 2005). IDRs are protein segments rich in hydrophilic, polar, and charged amino acids (glutamine, serine, glutamic acid, arginine, and lysine), as well as glycine, proline, and alanine (Iakoucheva *et al*., 2004; Liu *et al*., 2006). IDRs are prevalent in proteins that exhibit multiple physical interactions and play critical roles in various biological processes, including regulation and signaling (Dunker *et al*., 2000; Nguyen Ba *et al*., 2012; Forman-Kay and Mittag, 2013). The conformational flexibility of IDRs facilitates exposure of specific residues for modification and binding to other proteins and molecules (Huang and Sarai, 2012; Liu and Huang, 2014). Thus, intrinsically disordered proteins (IDPs) are characterized by a high IDR content and the absence of stable well-folded three-dimensional structures in solutions (Forman-Kay and Mittag, 2013).

Short linear motifs (SLIMs) are short stretches in protein sequences that mediate protein-protein interactions. SLIMs are typically 2–10 amino acids long; however, only two or three amino acids are essential for interaction with other molecules. SLIMs are common elements in IDRs, and they probably play a significant role in the functioning of these disordered regions (Wagner and Lynch, 2008; Huang and Sarai, 2012; Nguyen Ba *et al*., 2012; Forman-Kay and Mittag, 2013; Liu and Huang, 2014). The presence of a great number of these motifs in such regions probably confers functional flexibility to this class of proteins (Gould *et al*., 2010; Disfani *et al*., 2012; Dinkel *et al*., 2012, 2014). Furthermore, SLIMs are particularly evolvable because they are poorly conserved between lineages and can appear and disappear through small changes (Wagner and Lynch, 2008). Therefore, changes in SLIMs significantly impact complex regulatory networks (Neduva and Russell, 2005). Thus, analysis of these changes enables the assessment of their importance in the evolutionary trajectory of animals.

In addition to the forkhead, leucine-zipper, and zinc-finger domains, other molecular elements such as IDRs may play crucial roles in the function of FOXP proteins. However, these structures have not been studied extensively. Thus, the present investigation aims to ask how FOXPs structural forms changed throughout Tetrapoda evolution regarding linear motifs composition and disordered content. Furthermore, as *FOXP3* is known to be the only gene among the *FOXP* family playing a role in the immune system, we investigated if a higher evolutionary rate would be observed when compared with other *FOXP*s, and if such a rate could be related with higher disordered content.

## Material and Methods

Seventy-seven orthologues *FOXP* genes/proteins from tetrapods (Table S1) were considered in the present study. *FOXP* nucleotide sequences were retrieved from the NCBI database using BLASTN with 20,000 Max target sequences. We also used the Ensembl genome database (http://ensembl.org/) for sequence retrieval. The Neanderthal exome (Castellano *et al*., 2014; http://cdna.eva.mpg.de/) was consulted to verify possible specific changes within the genus *Homo*. However, one protein-coding gene may codify more than one isoform. The presence of many isoforms in the *FOX* genes, caused by alternative splicing, was handled conservatively by choosing only isoforms that clearly resemble the canonical form identified in humans by using UniProt (http://www.uniprot.org/). Incomplete sequences were removed from the analysis. Subsequently, the sequences were aligned using the MAFFT algorithms (standard pattern) implemented in the Guidance web server (http://guidance.tau.ac.il/). The alignments are available in the Supplementary Material. Phylogenetic trees were drawn using FigTree1.4.2. (http://tree.bio.ed.ac.uk/software/figtree/) according to the literature (Meredith *et al*., 2011; Perelman *et al*., 2011; Song *et al*., 2012).

Importantly, while both *FOXP2* and *FOXP4* passed through a standard NsSites test site analysis, for *FOXP3* and *FOXP1* we had to employ distinct data tests. Because of the absence of several base pairs in *Xenopus laevis* *FOXP1*, we excluded this species. For *FOXP3*, just the mammalian sequences were used because reptilian and amphibian *FOXP3* are shorter and very different, while in birds, *FOXP3* is completely absent (Andersen *et al*., 2012). In addition, we removed from the analysis a residual N-terminal part of *FOXP3* present only in the mammals *Nomascus leucogenys Papio anubis*, *Chlorocebus sabaeus, Callithrix jacchus*, *Cricetulus griseus*, *Panthera tigris*, *Myotis brandtii*, *Pteropus alecto*, *Chrysochloris asiatica*, and *Dasypus novemcinctus*, as they do not align or resemble other orthologous and known isoforms.

We predicted disordered regions by using the PONDR-FIT metapredictor (Xue *et al*., 2010a). Additionally, the MobiDB server (Potenza *et al*., 2014) was consulted to check consensus predictions for their disorder content, as provided by a variety of disorder predictors. SLIMs were predicted using the ELM webserver (Dinkel *et al*., 2012, 2014) considering only the cell nucleus as the cell compartment for biochemical interaction context of FOXP proteins. Given that the linear motifs predicted by ELM can present a high rate of false positives, we considered only ELM in IDR regions and validated such predictions by analyzing the literature on the interactions between linear motifs and their ligands with other transcription factors. Therefore, we considered only linear motifs with confirmed experimental data and/or certainty for ELM reliability annotation. All information regarding the linear motifs was retrieved from the ELM server and from the literature. The ELM server classifies SLIMs into the following four types: protease cleavage sites, protein motif interaction/binding sites, posttranslational modification sites, and subcellular targeting signals (Dinkel *et al*., 2012). Linear motifs present in the forkhead, leucine-zipper, and zinc-finger domains were not considered because they can represent false positives. Statistical tests comparing sites under purifying selection and/or positive selection within and without disordered regions were performed using WinPepi and SPSS 2.0.

To estimate the molecular evolutionary patterns of *FOXP1*, *FOXP2*, *FOXP3*, and *FOXP4*, we applied phylogeny-based maximum likelihood analysis of ω (non-synonymous/synonymous rate ratio or dN/dS) implemented in the PAML 4.7 package (Yang, 2007). This approach allows the ω ratio to vary among sites while considering several different codon substitution models. A value of ω < 1 indicates potential negative selection, while ω = 1 indicates neutrality, and ω > 1 indicates positive selection. For the NsSites codon substitution model, likelihood ratio tests (LRT) were performed between neutral models (M1a, nearly neutral, M8a, Beta and ω = 1) and models that allow positive selection and/or relaxation of functional constraints (M2a, positive selection and M8, Beta + Selection). Using log values from models M1a, M2a, M8a, and M8, we applied an LRT using HyPhy 2.2.0.

The Branch Site Model was also used to detect if different linear motif composition and disorder scores are reflected in different evolutionary rates among Tetrapoda. The phylogeny was a priori divided into two clades, and a LRT was used to evaluate divergences in selective pressures between them, as indicated by different ω ratios. We employed the clade model type D that assumes two site classes, which was compared with the neutral model M1a by an LRT with two degrees of freedom.

A Bayes empirical Bayes (BEB) approach was considered using CODEML in PAML 4.7 to verify which sites could be under neutral, purifying, or positive selection. The phylogenetic trees used to construct the PAML 4.7 input files were revised as described previously (Meredith *et al*., 2011; Perelman *et al*., 2011; Song *et al*., 2012).

## Results and Discussion

### FOXP1, FOXP2, FOXP3 and FOXP4 structures and their intrinsic protein disorder content

Our analyses revealed that the three paralogous proteins with similar functions and tissue expression, FOXP1, FOXP2, and FOXP4, had high and similar disorder contents (~70%). In contrast, FOXP3, which plays a role in immune system regulation, presented a lower disorder degree (~30%) relative to its paralogs (Tables 1, S2-S5), according to PONDR-FIT. The patterns of the disordered and ordered regions, as well as the disorder proportion of orthologous proteins, are relatively conserved among taxonomic groups (Tables 1, S2-S5). However, mammals presented a higher degree of protein disorder than all other organisms for FOXP1 and FOXP4 (*P* < 0.001, Table 1). Particularly, amphibians presented a lower degree of disorder for FOXP2 (~64%, Tables 1 and S3.1) than the other classes (*P* < 0.01, Table 1). These *FOXP* disorder prediction values are, in general, higher than those obtained by other authors (Andersen *et al*., 2012), but they used just partial proteins and fewer species. Importantly, it is worthy of note that the larger mammalian sample compared to non-mammals may have contributed to these statistical differences in the protein disorder content analysis.

**Table 1:** Mean disorder proportion for FOXP proteins by class^1^.

Class	FOXP2	FOXP4	FOXP1	FOXP3^2^
Mammals	0.7011	0.7321	0.6915	0,3065
Birds	0.7039	0.6858	0.6782	
Reptiles	0.6984	0.6827	0.6713	
Amphibians	0.6305	0.7068	NA	

1 Mammals showed significant higher proportions than the other groups, as assessed by the Kruskal-Wallis test, for FOXP1and FOXP4 (*P* < 0.001). Additionally, according to the same test, amphibians presented a lower degree of disorder for FOXP2 (*P* < 0.01).

2 Only mammalian genes were used for the FOXP3 analysis.

NA: Not available. Since several base pairs in *Xenopus laevis* FOXP1 sequence are missing, we excluded it from the analysis.

Interestingly, our data reveals that mammals present significantly higher FOXP1 and FOXP4 disorder degrees than the other groups. This finding may be associated with the more complex interaction networks present in mammals, as already proposed for other genetic systems (Disfani *et al*., 2012), and to a positive correlation between the number of binding partners and disorder scores (Dunker *et al*., 2000). Thus, it is reasonable to speculate that mammalian FOXP1 and FOXP4 present a larger number of binding partners than the other orthologues investigated here.

### FOXP1, FOXP2, FOXP3, and FOXP4 and their interaction sites

Usually, intrinsically disordered proteins are enriched with SLIMs, which play crucial roles in their interaction with other proteins (Tables S6.1-6.4). Here we will briefly describe some selected representative results of the SLIMs compositional analysis. For FOXP1, some of our findings include a Polo-like kinase 1 (PLK) phosphorylation site at position 33 (MOD_PLK), which differentiates Sauropsida (reptiles and birds) from mammals (Table 2). PLK is involved in cell cycle events (Nakajima *et al*., 2003; Murakami *et al*., 2010), suggesting some differences in the FOXP1 phosphorylation pattern during the cell cycle between mammals and Sauropsida.

**Table 2:** Linear motifs changes in representative species of *Tetrapoda*, as predicted by ELM.

Aligned Position		Nucleotide	Amino acid	Grantham Score	*Homo sapiens*	*Pan troglodytes*	*Pan paniscus*	*Mus musculus*	*Taeniopygia guttata*	*Serinus canaria*	*Anolis carolinensis*	*Xenopus laevis*
FOXP1	33	GGT- > AGT	Gly- > Ser	56	0	0						*
		GGT- > GCA	Gly- > Ala	60				1				
		GGT- > AGC	Gly- > Ser	56					1^p,r,v^	1^p,r,v^		
		GGT- > GGC	Gly- > Gly	Syn							1^p,r,v^	
FOXP2	314	GCA- > GCG	Ala- > Ala	Syn	0	0		0				
		GCG- > CCA	Ala- > Pro	27								
		GCG- > TCT	Ala- > Ser	99					1^d^	1^d^	1^d^	1^d,s^
		GCG- > CCC	Ala- > Pro	27								
	368	AAC- > ACC	Asn- > Thr	65	0^o4,q3^	1^o4,q4^		1^o4,q4^	1^o4,q4^	1^o4,q4^	1^o4,q4^	1^o4,q4^
	390	AGT- > AAT	Ser- > Asn	46	0^o,q^	1		1	1	1	1	0^o^
FOXP4	408	CCG- > CCA	Pro- > Pro	Syn	0		0	1				
		CCG- > CTG	Pro- > Leu	98					1^i^	1^i^	1^i^	
		CCG- > TTG	Pro- > Leu	98								1^i^
	689	TCG- > TCA	Ser- > Ser	Syn	0^d^		0^d^					
		TCG- > TTG	Ser- > Leu	145								
		TCG- > GTG	Ser- > Val	124				1				
		TCG- > ACA	Ser- > Thr	58							1^k^	
		TCG- > ACG	Ser- > Thr	58					1^k^	1^k^		
		TCG- > GTC	Ser- > Val	124								1^j^

* indicates gap.

Syn = synonymous changes.

Zero (0) indicates the amino acid present in the *Homo sapiens* reference sequence, whereas 1 indicates a variant amino acid .Subscribed letters indicate the predicted presence of specific Eukaryotic Linear Motifs (see code shown in Table S9). Subscribed numbers are the number of times that each SLIMs appeared.

The nature of modification is not representing an ancestry and descendant relationship. Grantham scores predicted as conservative (0-50), moderately conservative (51-100), moderately radical (101-150) or radical (> 151).

In the case of FOXP2 (Table 2), mammals have lost one DOC_USP7_1 at position 314, which interacts with the deubiquitinating enzyme USP7/HAUSP (herpes virus-associated ubiquitin-specific protease) present in all non-mammals, due to a serine to alanine change. Previous studies have demonstrated that the interaction of USP7 with FOX members regulates oxidative stress responses through ubiquitination (van der Horst *et al*., 2006). Thus, the possible loss of DOC_USP7_1 in mammals could have a functional implication related to response to oxidative stress.

Two known non-synonymous substitutions between humans (*Homo sapiens* and Neanderthals) and chimpanzees (FOXP2 Asn325Ser and Thr303Asn) deserve additional attention, since they were related to human speech (Enard *et al*., 2002, Krause *et al*., 2007). One of them (Asn325Ser) promotes the gain of two motifs, MOD_CK1_1 and MOD_GSK3_1, in humans due to the presence of a serine at aligned position 390 (Table 2). Both motifs are promoters of phosphorylation by kinases. Interestingly, carnivores also have a serine at this FOXP2 ortholog position (Zhang *et al*., 2002), leading to a convergence event of the emergence of both MOD_CK1_1 and MOD_GSK3_1 motifs observed in humans. Cooper (2006) suggested that phosphorylation by kinase C in this FOXP2 region may be related to human behavioral traits such as language. However, the other *Homo-*specific substitution at aligned position 368 (Thr303Asn) led to the loss of a phosphorylation site. Changes in phosphorylation patterns can modulate the regulation of transcription factors and their binding affinity to co-activators and DNA. These changes can in turn alter gene expression, cell growth, and differentiation (Iakoucheva *et al*., 2004). Thus, our results have one very relevant implication: the loss of this phosphorylation site at position 368/303 can have been as important as the gain of the phosphorylation site at position 390/325 for the evolution of human speech. The phenotype implication of the presence of these SLIMS in carnivores is unknown.

For FOXP3, which was only investigated in mammals (see Material and Methods section), a CK1 phosphorylation site (MOD_CK1_1) is predicted at position 194 (Table 3) for several mammal species, except New World (NW) monkeys (*Saimiri boliviensis* and *Callithrix jacchus*) and *Tarsius syrichta*. Interestingly, these primates present four other linear motifs in this region: MOD_GSK3_1, MOD_ProDkin_1, DEG_SCF_FBW7_1, and DOC_WW_Pin1_4. Therefore, we identified the presence of the same SLIMs in two distinct branches of primates (New World monkeys and Tarsiidae) that live in somewhat similar rainforest environments. As mentioned before, FOXP3 is the only FOXP member playing a role in the immune system, suggesting that at least one of these motifs is associated with the immune response, indicating adaptation through convergence or the maintenance of a primate ancestral state.

**Table 3:** FOXP3 Linear motifs changes in Mammals, as predicted by ELM.

Aligned Position	194						
Nucleotide	GTG- > ATG	GTG- > ACA	GTG- > TTG	GTG- > GGG	GTG- > GCA	GTG- > GCG	GTG- > ACG
Amino acid	Val- > Met	Val- > Thr	Val- > Leu	Val- > Gly	Val- > Ala	Val- > Ala	Val- > Thr
Grantham Score	21	69	32	109	64	64	69
*Homo sapiens*	0^o^						
*Pan troglodytes*	0^o^						
*Pan paniscus*	0^o^						
*Gorilla gorilla*	0^o^						
*Pongo abellii*	0^o^						
*Pongo pygameus*	0^o^						
*Hylobates lar*	0^o^						
*Nomascus*	0^o^						
*Macaca mulatta*	1^o^						
*Papio anubis*	1^o^						
*Chlorocebus sabaeus*	1^o^						
*Saimiri boliviensis*		1^b e o q2 w^					
*Callithrix jacchus*		1^b e o q2 w^					
*Galeopterus variegatus*	0^o^						
*Tarsius syrichta*		1^b e o q2 w^					
*Tupaia chinensis*			1^m3 j o^				
*Sorex araneus*				1^o^			
*Mus musculus*					1^c^		
*Cricetulus griseus*					1^c^		
*Rattus norvegicus*					1^c^		
*Octodon degus*	1^o^						
*Oryctolagus cuniculus*						1^o^	
*Ochotona princeps*						1^o^	
*Physeter catodon*	1^o^						
*Orcinus orca*	1^o^						
*Camelus ferus*	0^o^						
*Bos taurus*	0^o^						
*Equus caballus*	1^o^						
*Ailuropoda melanoleuca*	0^o^						
*Felis catus*	0^o^						
*Canis lupus familiaris*	0						
*Vicugna pacos*	0^o^						
*Panthera tigris*	0^o^						
*Mustela putorius furo*	0^o^						
*Odobenus rosmarus*	0^o^						
*Leptonychotes weddellii*	0^o^						
*Ceratotherium simum*							1^b e o q2 w^
*Eptesicus fuscus*	0^o^						
*Myotis brandtii*	0^o^						
*Pteropus alecto*	1^s^						
*Condylura cristata*						1^o^	
*Chrysochloris asiatica*	1^c^						
*Erinaceus europaeus*	1						
*Echinops telfairi*							1^b e o q2 w^
*Orycteropus afer afer*							1^b e o q2 w^
*Loxodonta africana*	1^f t v^						
*Trichechus manatus*	1^e w^						
*Dasypus novemcinctus*						1^o2 s^	

* indicates gap. Syn = synonymous change. Zero (0) indicates the amino acid present in the *Homo sapiens* reference sequence whereas 1 indicates a variant amino acid .Subscribed letters indicate the predicted presence of specific Eukaryotic Linear Motifs (see code shown in Table S9). The nature of modification is not representing an ancestry and descendant relationship. Grantham scores predicted as conservative (0-50) moderately conservative (51-100) moderately radical (101-150) or radical (> 151).

Another interesting finding is the sharing of the linear motif LIG_PTAP_UEV_1 between Neanderthals and modern humans due to the Gly175Ser (human position) mutation (Table 4). It has also been suggested that linear motifs mediate interactions between viruses with their hosts (Hagai *et al*., 2014). In fact, LIG_PTAP_UEV_1 mediates the binding of several cellular and viral proteins to the UEV domain of the class E vacuolar sorting protein Tsg101 (Göttlinger *et al*., 1991), and it is essential for the efficient egress of viral particles from many enveloped RNA viruses (Bieniasz, 2006). Our results indicate that this motif may have played an important role in *Homo* self-immune defense during the Pleistocene.

**Table 4:** FOXP3-specific changes in primates.

Organisms	Aligned position	Human position	AA Change	Motifs^1^
Neanderthal and Humans	140	132	Pro- > Thr	(+2) DEG_SCF_FBW7_1
	183	175	Gly- > Ser	(+) LIG_PTAP_UEV_1
				
Neanderthal	192	184	Ser- > Leu	(-) MOD_CK1_1, (+) DOC_MAPK_1
				
Catarrhini	278	270	Pro- > Ser	(+) MOD_GSK3_1
				
Haplorhini^2^	82	74	Val- > Leu	(-) DOC_WW_Pin1_4, (-)MOD_ProDKin_1
	97	89	Ser- > Leu	
	129	121	Arg- > His	
	132	124	Asp- > Glu	
	181	173	Ser- > Asn	(-)DOC_WW_Pin1_4, (-) MOD_ProDKin_1
	246	238	Val- > Met	
	262	254	Gly- > Ser	
	338	325	Phe- > Leu	
	424	411	Phe- > Leu	

1 +: change causes motif gain; -: change causes motif loss.

2 Excluding *Tarsius syrichta*.

Regarding *FOXP4*, a striking difference between mammals and Sauropsida was also found (birds and reptiles, Table 2). For instance, the loss of LIG_CtBP_PxDLS_1 in mammals is due to the substitution of a leucine for proline at aligned position 408, probably after the divergence of Synapsida and Sauropsida. Mendoza *et al*. (2015) showed that the presence of the CtBP binding region in the bird *Taeniopygia guttata* has been associated with the potential FOXP4 regulation capacity. This finding for CtBP interaction may be associated with an enhanced potential for transcriptional repression of FOXP4, known for FOXP1 and FOXP2 (Mendoza *et al*., 2015). At aligned position 689, almost all non-mammals present a motif that interacts with FHA (LIG_FHA_1 or LIG_FHA_2), while mammals present a DOC_USP7_1 motif.

To better understand the role of SLIMs in evolution, we additionally compared members within the *FOXP* family to verify the number of unique linear motifs in each paralog (Table 5). The number of predicted types of SLIMs range from 28 (FOXP2) to 39 (FOXP4). Furthermore, FOXP3 presents three unique motifs (DOC_PP2B_1, TRG_NLS_MonoCore_2, and TRG_NLS_MonoExtN_4), FOXP1 presents four (DEG_SCF_FBW7_2, LIG_PCNA_PIPBox_1, LIG_WD40_WDR5_1, and TRG_NES_CRM1_1), while FOXP2 presents no unique SLIM. FOXP4 presents six motifs, among which three (DOC_PP1_RVXF_1, LIG_BRCT_BRAC1_2, and TRG_NLS_MonoExtC_3) are common to almost all species investigated in the current study.

**Table 5:** Number of shared and unique short linear motifs (SLIMs) among Tetrapoda FOXPs.

Protein	Total type of SLIMs	Number of unique SLIMs	Total SLIMs in *Homo sapiens*	Total SLIMs in *Pan sp*.	Total SLMIs in *Serinus canaria* 1	Total of species compared
FOXP1	34	4^2^	132	132	135	50
FOXP2	28	0	143	142	140	54
FOXP3	32	3^3^	69	62	-	57
FOXP4	39	6^4^	142	143	160	65

1 Bird, representing Sauropsida.

2 DEG_SCF_FBW7_2, LIG_PCNA_PIPBox_1, LIG_WD40_WDR5_1, and TRG_NES_CRM1_1);

3 DOC_PP2B_1, TRG_NLS_MonoCore_2, and TRG_NLS_MonoExtN_4;

4 FOXP4 presents six motifs, among which three (DOC_PP1_RVXF_1, LIG_BRCT_BRAC1_2, and TRG_NLS_MonoExtC_3) are common to almost all species investigated in the current study.

### Molecular evolutionary patterns

Evolutionary tests for *FOXP1, FOXP2, FOXP3* and *FOXP4* considering all the tetrapod species investigated in this study indicated that the best log-likelihood model is M1a, which assumes purifying selection and neutral ω values. *FOXP1*, *FOXP2* and *FOXP4* present more than 95% of the sites, with ω = 0.03066, 0.01965 and 0.02778, respectively (Table S7), indicating a strong role for purifying selection. *FOXP3* presents 10% of ω values equal to 1, which indicates molecular neutral evolution and/or relaxation of functional constraints.

Additionally, we used the results from the Bayes Empirical Bayes (BEB) test to calculate the posterior probabilities that each codon is under positive selection (Yang, 2007). The BEB values are only significant for M2 and M8 (that include such selection), therefore this last strategy was only adopted to detect eventual functional sites. Such analysis showed four sites in mammals with ω > 1 and probability > 91%, but the *p* value was not significant (Table S7). Regardless, it is important to highlight that one of the sites inferred with ω = 1.06 (probability = 98.9%) is located at position 194 of *FOXP3* (Table 3). This position presents differences in SLIM prediction (MOD_GSK3_1, MOD_ProDkin_1, MOD_CK1_1, DEG_SCF_FBW7_1, and DOC_WW_Pin1_4) in *Saimiri boliviensis, Callithrix jacchus*, and *Tarsius syrichta* when compared with the other species. *Saimiri boliviensis* and *Callithrix jacchus* probably share the same linear motifs because of their clear and relatively recent common origin, but *Tarsius syrichta*, which is phylogenetically more distant, may present them because of convergent evolution (Tables S6.3 and 3). MOD_ProdKin_1 is a post-translational modification site phosphorylated by a MAP kinase, while DEG_SCF_FBW7_1 is a degradation site mediated by an important protein complex (Skp, Cullin, F-box containing complex or SCF) that plays a role in checkpoints during the cell cycle (Nguyen Ba *et al*., 2012). DOC_WW_Pin1_4 interacts with the enzyme Pin1, whose function is also associated with the cell cycle, among others. Additionally, Pin1 regulates the immune response (Gavva *et al*., 1997; Wulf *et al*., 2002; Wijchers *et al*., 2006; Saxena *et al*., 2010), which is a known function of FOXP3. Again, as we identified the presence of the same SLIMs in two distinct primate branches that live in similar environments (rainforest), this allow us to infer that a simple neutral model is insufficient to explain this scenario.

In the case of *FOXP4* (Table S8), the Branch Site model indicated that mammals have a ω value 3.7 times higher than non-mammals (0.66102 *versus* 0.18012), a result compatible with relaxation of evolutionary pressures. This striking difference (*p* < 0.001) may be attributed to certain changes, such as the absence of the interaction site for CtBP (LIG_CtBP_PxDLS_1) in all mammals (except *Sus scrofa*). Another structural/functional change that can explain the distinct ω values observed between mammals and non-mammals is the presence of a glutamine-rich region in mammalian FOXP4, associated to its repression ability.

## Conclusion

Our study reveals some important general and more specific findings. For instance, 70% of the disorder content has been retained in FOXP1, FOXP2, and FOXP4 orthologs. Some of the results obtained can be associated with taxa-specific conditions, while others may represent molecular convergence. In fact, we found changes at FOXP3 sites with possible functional implications in the primate branch, including the genus *Homo*. Finally, the FOXP1 and FOXP4 results show instigating differences between mammals and non-mammals, suggesting their role in the emergence of adaptive novelty within the taxon Tetrapoda. Our results indicate that part of the *FOXP* evolutionary “stability” over a long evolutionary period may be attributed to the maintenance of a similar proportion of disordered regions, but not to amino acid content or linear motifs. Moreover, some of the changes can be interpreted as indicating taxa-specific adaptations, since they are probably functional.

## Supplemental Material

The following online material is available for this article:

Table S1 - List of Tetrapoda species and the respective sequence codes retrieved for each of the FOXP subfamily members studied.

Table S2.1 - Disorder proportion of FOXP1 orthologues.

Table S2.2 - Ordered regions of FOXP1 orthologues.

Table S3.1 - Disorder proportion of FOXP2 orthologues.

Table S3.2 - Ordered regions of FOXP2 orthologues.

Table S4.1 - Disorder proportion of FOXP3 orthologues.

Table S4.2 - Ordered regions of FOXP3 orthologues.

Table S5.1 - Disorder proportion of FOXP4.

Table S5.2 - Ordered regions of FOXP4.

Table S6.1 - Whole protein comparison for FOXP1 linear motifs content.

Table S6.2 - Whole protein comparison for FOXP2 linear motifs content.

Table S6.3 - Whole protein comparison for FOXP3 linear motifs content.

Table S6.4 - Whole protein comparison for FOXP4 linear motifs content.

Table S7 - Estimated parameters under different codon substitution models for forkhead P subfamily genes.

Table S8 - Branch site model for *FOXP* genes.

Table S9 - Eukaryotic linear motifs and their functions.
