# Supplementary material for: FOXP in Tetrapoda: Intrinsically Disordered Regions, Short Linear Motifs and their evolutionary significance
Source: Genet Mol Biol. 2017 Mar 2;40(1):181–90. doi: 10.1590/1678-4685-GMB-2016-0115 (PMC5409772; doi:10.1590/1678-4685-GMB-2016-0115)
Supplement: Supplementary file 1 [file 1415-4757-gmb-1678-4685-GMB-2016-0115-Suppl01.pdf]

Table S1. List of Tetrapoda species and the respective sequence codes retrieved to each one of the FOXP subfamily member studied.

Class	Common Name	Species	*FOXP1*	*FOXP2*	*FOXP3*	*FOXP4*
Mammalia	Human	*Homo sapiens*	NM_032682.5[a]	ENST00000350908[b]	ENST00000376207[b]	ENST00000373060[b]
Mammalia	Chimpanzee	*Pan troglodytes*	XM_001140515.3[a]	ENSPTRT00000036314 b	JX296015.1a	ENSPTRT00000042554.3 b
Mammalia	Bonobo	*Pan paniscus*	-	-	XM_003807259.2[a]	XM_003833311.2[a]
Mammalia	Gorilla	*Gorilla gorilla gorilla*	XM_004035888.1[a]	AY143180.1[a]	ENSGGOT00000025034 b	ENSGGOT00000016440[b]
Mammalia	Orangutan	*Pongo abelii*	XM_009238796.1[a]	ENSPPYT00000020887[b]	ENSPPYG00000020341[b]	XM_002816867.3[a]
Mammalia	Pygmy Orangutan	*Pongo pygmaeus*	-	-	JX296014.1[a]	-
Mammalia	Gibbon	*Nomascus leucogenys*	XM_003264887.2[a]	XM_003261215.2[a]	ENSNLET00000010324[b]	XM_003266292.1[a]
Mammalia	White-hand Gibbon	*Hylobates lar*	-	-	JX296016.1[a]	-
Mammalia	Macaque	*Macaca mulatta*	NM_001266321.1[a]	NM_001033021.1[a]	NM_001032918.1[a]	NM_001266091.1[a]
Mammalia	Baboon	*Papio anubis*	XM_009200345.1[a]	XM_009203721.1[a]	ENSPANT00000010875[b]	ENSPANT00000017705[b]
Mammalia	Vervet monkey	*Chlorocebus sabaeus*	XM_007984970.1[a]	XM_007982653.1[a]	XM_007991691.1[a]	XM_007972623.1[a]
Mammalia	Squirrel monkey	*Saimiri boliviensis boliviensis*	XM_003939460.1[a]	XM_003921066.1[a]	XM_010350232.1[a]	XM_003922987.1[a]
Mammalia	Marmoset	*Callithrix jacchus*	XM_002758548.3[a]	XM_002751708.3[a]	GQ284839.1[a]	XM_003732665.2[a]
Mammalia	Sunda flying lemur	*Galeopterus variegatus*	XM_008571774.1[a]	XM_008571504.1[a]	XM_008587131.1[a]	XM_008583550.1[a]
Mammalia	Bushbaby	*Otolemur_garnettii*	-	-	-	ENSOGAT00000010116[b]
Mammalia	Tarsier	*Tarsius syrichta*	-	-	XM_008074655.1[a]	XM_008062590.1[a]
Mammalia	Shrew	*Sorex araneus*	-	-	XM_004606453.1[a]	XM_004605787.1[a]
Mammalia	Chinese tree Shrew	*Tupaia chinensis*	XM_006158050.1[a]	XM_006157413.1[a]	XM_006163498.1[a]	XM_006148586.1[a]
Mammalia	Mouse	*Mus musculus*	ENSMUST00000113326 b	NM_053242.4[a]	NM_001199348.1[a]	ENSMUST00000097311[b]
Mammalia	Rat	*Rattus norvegicus*	ENSRNOT00000013271[b]	XM_006236115.2[a]	ENSRNOT00000015641[b]	XM_008766868.1[a]
Mammalia	Chinese hamster	*Cricetulus griseus*	XM_007631502.1[a]	XM_007650925.1[a]	XM_007630484.1[a]	XM_003508987.2[a]
Mammalia	Degu	*Octodon degus*	XM_004629502.1[a]	XM_004644864.1[a]	XM_004646275.1[a]	XM_004624157.1[a]
Mammalia	Guinea_pig	*Cavia_porcellus*	-	-	-	XM_003473873.2[a]

Database: a, NCBI; b Ensemble, *individuals used just for disorder analysis.

Table S1. List of Tetrapoda species and the respective sequence codes retrieved to each one of the FOXP subfamily member studied (continued).

Class	Common Name	Species	*FOXP1*	*FOXP2*	*FOXP3*	*FOXP4*
Mammalia	Rabbit	*Oryctolagus cuniculus*	XM_002713312.2[a]	NM_001122936.1[a]	ENSOCUT00000010135[b]	-
Mammalia	Pika	*Ochotona princeps*	XM_004581620.1[a]	XM_004592462.1[a]	XM_004587932.1[a]	XM_004590349.1[a]
Mammalia	Sperm whale	*Physeter catodon*	XM_007105389.1[a]	XM_007106509.1[a]	XM_007113265.1[a]	XM_007120287.1[a]
Mammalia	Kiler whale	*Orcinus orca*	-	-	XM_004281924.1[a]	XM_004267622.1[a]
Mammalia	Camel	*Camelus ferus*	-	XM_006184670.1[a]	XM_006195238.1[a]	XM_006202021.1[a]
Mammalia	Alpaca	*Vicugna_pacos*	XM_006196491.1[a]	XM_006206156.1[a]	XM_006213486.1[a]	XM_006202021.1[a]
Mammalia	Cow	*Bos taurus*	-	-	NM_001045933.1[a]	NM_001205524.1[a]
Mammalia	Horse	*Equus caballus*	-	-	NM_001163272.1[a]	ENSECAT00000001439[b]
Mammalia	Pig	*Sus_scrofa*	-	-	-	XM_001926847.2[a]
Mammalia	Weddell seal	*Leptonychotes weddellii*	-	-	XM_006743003.1[a]	XM_006729196.1[a]
Mammalia	Walrus	*Odobenus rosmarus divergens*	XM_004404552.1[a]	XM_004408502.1[a]	XM_004396823.1[a]	XM_004408054.1[a]
Mammalia	Panda	*Ailuropoda melanoleuca*	-	-	XM_002917744.1[a]	XM_002914517.1[a]
Mammalia	Cat	*Felis catus*	-	NM_001113177.1[a]	ENSFCAT00000003824[b]	-
Mammalia	Tiger	*Panthera tigris*	-	XM_007089152.1[a]	XM_007095999.1[a]	-
Mammalia	Ferret	*Mustela_putorius_furo*	-	-	ENSMPUT00000014169[b]	XM_004739938.1[a]
Mammalia	Dog	*Canis lupus familiaris*	-	-	AB501357.1[a]	XM_538914.5[a]
Mammalia	Rhinoceros	*Ceratotherium simum simum*	-	XM_004418795.1[a]	XM_004434916.1[a]	XM_004424166.1[a]
Mammalia	Brandt's myotis	*Myotis brandtii*	XM_005885064.1[a]	XM_005862259.1[a]	XM_005864530.1[a]	XM_005885813.1[a]
Mammalia	Big brown bat	*Eptesicus fuscus*	XM_008154141.1[a]	XM_008152407.1[a]	XM_008158246.1[a]	XM_008158925.1[a]
Mammalia	Black flying fox	*Pteropus alecto*	XM_006917473.1[a]	XM_006910602.1[a]	XM_006922727.1[a]	XM_006926566.1[a]
Mammalia	Star-nosed mole	*Condylura cristata*	XM_004675975.1[a]	XM_004676957.1[a]	XM_004690039.1[a]	XM_004673459.1[a]
Mammalia	Western European hedgehog	*Erinaceus europaeus*	XM_007523308.1[a]	XM_007516144.1[a]	XM_007529426.1[a]	-
Mammalia	Elephant-shrew	*Elephantulus edwardii*	XM_006900488.1[a]	XM_006882655.1[a]	XM_006902719.1[a]	XM_006882009.1[a]

Database: a, NCBI; b Ensemble, *individuals used just for disorder analysis.

Table S1. List of Tetrapoda species and the respective sequence codes retrieved to each one of the FOXP subfamily member studied (continued).

Class	Common Name	Species	*FOXP1*	*FOXP2*	*FOXP3*	*FOXP4*
Mammalia	Cape golden mole	*Chrysochloris asiatica*	XM_006874623.1[a]	XM_006859303.1[a]	XM_006876686.1[a]	XM_006860443.1[a]
Mammalia	Lesser hedgehog tenrec	*Echinops telfairi*	XM_004702425.1[a]	XM_004702573.1[a]	XM_004713140.1[a]	-
Mammalia	Aardvark	*Orycteropus afer afer*	XM_007946080.1[a]	XM_007944086.1[a]	XM_007958374.1[a]	XM_007936267.1[a]
Mammalia	Mannatee	*Trichechus manatus latirostris*	XM_004372708.1[a]	XM_004382570.1[a]	XM_004376834.1[a]	XM_004379437.1[a]
Mammalia	Elephant	*Loxodonta africana*	XM_003409786.1[a]	XM_003407220.1[a]	ENSLAFT00000003503[b]	XM_003403923.1[a]
Mammalia	Armadillo	*Dasypus novemcinctus*	-	-	XM_004465020.1[a]	-
Mammalia	Opossum	*Monodelphis domestica*	XM_007500090.1[a]	XM_007504167.1[a]	-	-
Mammalia	Platypus	*Ornithorhynchus_anatinus*	-	-	XM_001507231.3[a]	-
Bird	Duck	*Anas platyrhynchos*	ENSAPLT00000012301[b]	XM_005010980.1[a]	-	XM_005023422.1[a]
Bird	Emperor penguin	*Aptenodytes forsteri*	XM_009284364.1[a]	XM_009286822.1[a]	-	XM_009272821.1[a]
Bird	Anna's hummingbird	*Calypte anna*	XM_008499642.1[a]	XM_008496149.1[a]	-	XM_008499441.1[a]
Bird	Peregrine falcon	*Falco peregriunus*	XM_005229951.1[a]	XM_005242504.1[a]	-	XM_005234510.1[a]
Bird	Flycatcher	*Ficedula albicollis*	XM_005053288.1[a]	XM_005039338.1[a]	-	ENSFALT00000003213[b]
Bird	Chicken	*Gallus gallus*	NM_001024827.1[a]	ENSGALT00000015345[b]	-	XM_003642748.2[a]
Bird	Golden-collared manakin	*Manacus vitellinus*	XM_008925680.1[a]	XM_008932480.1[a]	-	XM_008934298.1[a]
Bird	Budgerigar	*Melopsittacus undulatus*	XM_005149418.1[a]	NM_001281546.1[a]	-	XM_005142863.1[a]
Bird	Ground tit	*Pseudopodoces humilis*	XM_005521985.1[a]	-	XM_005533346.1[a]	XM_005529345.1[a]
Bird	Canary	*Serinus canaria*	XM_009091186.1[a]	XM_009086754.1[a]	-	XM_009098374.1[a]
Bird	Zebra finch	*Taeniopygia_guttata*	ENSTGUT00000010342[b]	NM_001048263.2[a]	-	NM_001279260.1[a]
Bird	White-throated sparrow	*Zonotrichia albicollis*	XM_005491385.1[a]	XM_005481975.1[a]	-	XM_005492836.1[a]
Reptile	Crocodile	*Alligator mississippiensis*	-	-	XM_006261276.1[a]	XM_006275827.1[a]
Reptile	Chinese Crocodile	*Alligator sinensis*	-	-	XM_014523895.1[a]	XM_006025064.1[a]
Reptile	Anole lizard	*Anolis carolinensis*	XM_008105381.1[a]	BK008623.1[a]	XM_008103924.1[a]	XM_008109698.1[a]

Database: a, NCBI; b Ensemble, *individuals used just for disorder analysis.

Table S1. List of Tetrapoda species and the respective sequence codes retrieved to each one of the FOXP subfamily member studied (continued).

Reptile	Western Painted Turtle	*Chrysemys picta bellii*	-	-	-	XM_005308300.2[a]
Reptile	Green Turtle	*Chelonia mydas*	XM_007061459.1[a]	XM_007070534.1[a]	-	XM_007068896.1[a]
Reptile	Chinese softshell turtle	*Pelodiscus_sinensis*	ENSPSIT00000009876[b]	-	-	ENSPSIT00000013410[b]
Reptile	Python	*Python bivittatus*	XM_007421309.1[a]	XM_007428286.1[a]	XM_007420528.1[a]	XM_007426185.1[a]
Amphibious	Xenopus (silurana)	*Xenopus tropicalis*	-	-	XM_002940971.2[a]	NM_001077187.2[a]
Amphibious	Xenopus	*Xenopus laevis*	NM_001095533.1[a]	NM_001095669.1[a]	NM_001127727.1[a]	NM_001095615.1[a]
Amphibious	Emei music-frog	*Babina daunchina**	-	JX543513.1[a]	-	-
Amphibious	Paddle-tail newt	*Pachytriton labiatus**	-	JX543512.1[a]	-	-

Database: a, NCBI; b Ensemble, *individuals used just for disorder analysis.
