# Supplementary material for: FOXP in Tetrapoda: Intrinsically Disordered Regions, Short Linear Motifs and their evolutionary significance
Source: Genet Mol Biol. 2017 Mar 2;40(1):181–90. doi: 10.1590/1678-4685-GMB-2016-0115 (PMC5409772; doi:10.1590/1678-4685-GMB-2016-0115)
Supplement: Supplementary file 2 [file 1415-4757-gmb-1678-4685-GMB-2016-0115-Suppl02.pdf]

Table S2.1 Disorder proportion of FOXP1 orthologues.

Species	Disorder Proportion	AA	Order	Class
Homo sapiens	0.692762186	677	Primates	Mammal
Pan troglodytes	0.692762186	677	Primates	Mammal
Gorilla gorilla	0.692762186	677	Primates	Mammal
Pongo abelii	0.692762186	677	Primates	Mammal
Nomascus leucogenys	0.692762186	677	Primates	Mammal
Macaca mulatta	0.692762186	677	Primates	Mammal
Papio anubis	0.692762186	677	Primates	Mammal
Chlorocebus sabaeus	0.692762186	677	Primates	Mammal
Callithrix jacchus	0.692762186	677	Primates	Mammal
Saimiri boliviensis	0.687869822	676	Primates	Mammal
Galeopterus variegatus	0.666174298	677	Dermoptera	Mammal
Tupaia chinensis	0.690828402	676	Scandentia	Mammal
Mus musculus	0.694814815	675	Rodentia	Mammal
Rattus norvegicus	0.703234880	711	Rodentia	Mammal
Cricetulus griseus	0.692532943	683	Rodentia	Mammal
Octodon degus	0.702346041	682	Rodentia	Mammal
Oryctolagus cuniculus	0.697810219	685	Lagomorpha	Mammal
Ochotona princeps	0.696035242	681	Lagomorpha	Mammal
Physeter catodon	0.682020802	673	Cetacea	Mammal
Vicugna pacos	0.692762186	677	Artiodactyla	Mammal
Camelus ferus	0.622974963	679	Artiodactyla	Mammal
Ceratotherium simum simum	0.620029455	679	Perissodactyla	Mammal
Felis catus	0.622974963	679	Carnivora	Mammal
Panthera tigris	0.622974963	679	Carnivora	Mammal
Odobenus rosmarus divergens	0.685376662	677	Carnivora	Mammal
Chrysochloris asiatica	0.690615836	682	Afrosoricida	Mammal
Condylura cristata	0.688693098	681	Soricomorpha	Mammal
Echinops telfairi	0.689807976	677	Afrosoricida	Mammal
Elephantulus edwardii	0.701317716	683	Macroscelidea	Mammal
Eptesicus fuscus	0.693333333	675	Chiroptera	Mammal
Myotis brandtii	0.701624815	677	Chiroptera	Mammal
Pteropus alecto	0.671111111	675	Chiroptera	Mammal
Erinaceus europaeus	0.689910979	674	Erinaceomorpha	Mammal
Monodelphis domestica	0.676514032	677	Didelphimorphia	Mammal
Orycteropus afer afer	0.691285081	677	Tubulidentata	Mammal
Loxodonta africana	0.700292398	684	Proboscidea	Mammal
Trichechus manatus latirostris	0.697810219	685	Sirenia	Mammal
Serinus canaria	0.687134503	684	Passeriformes	Bird
Taeniopygia guttata	0.653521127	710	Passeriformes	Bird
Manacus vitellinus	0.682748538	684	Passeriformes	Bird
Ficedula albicollis	0.678885630	682	Passeriformes	Bird
Melopsittacus undulatus	0.680819912	683	Psittaciformes	Bird
Zonotrichia albicollis	0.681286550	684	Passeriformes	Bird
Falco peregrinus	0.671052632	684	Falconiformes	Bird

Table S2.1 Disorder proportion of FOXP1 orthologues (continued).

Species	Disorder Proportion	AA	Order	Class
Aptenodytes forsteri	0.670571010	683	Sphenisciformes	Bird
Calypte anna	0.689450223	673	Trochiliformes	Bird
Gallus gallus	0.670553936	686	Galliformes	Bird
Anas platyrhynchos	0.693997072	683	Anseriformes	Bird
Python bivittatus	0.678311499	687	Squamata	Reptilia
Anolis carolinensis	0.701601164	687	Squamata	Reptilia
Pelodiscus sinensis	0.649851632	674	Testudines	Reptilia
Chelonia mydas	0.655325444	676	Testudines	Reptilia
Xenopus laevis	0.600346021	578	Anura	Amphibia
