# Supplementary material for: FOXP in Tetrapoda: Intrinsically Disordered Regions, Short Linear Motifs and their evolutionary significance
Source: Genet Mol Biol. 2017 Mar 2;40(1):181–90. doi: 10.1590/1678-4685-GMB-2016-0115 (PMC5409772; doi:10.1590/1678-4685-GMB-2016-0115)
Supplement: Supplementary file 3 [file 1415-4757-gmb-1678-4685-GMB-2016-0115-Suppl08.pdf]

Table S5.1. Disorder proportion for FOXP4.

Species	Disorder Proportion	AA	Order	Class
Homo sapiens	0.726470588	680	Primates	Mammal
Pan paniscus	0.726470588	680	Primates	Mammal
Gorilla gorilla	0.726470588	680	Primates	Mammal
Pongo abelli	0.726470588	680	Primates	Mammal
Nomascus leucogenys	0.730882353	680	Primates	Mammal
Macaca mulatta	0.735294118	680	Primates	Mammal
Papio anubis	0.735294118	680	Primates	Mammal
Chlorocebus sabaeus	0.735294118	680	Primates	Mammal
Saimiri boliviensis boliviensis	0.733823529	680	Primates	Mammal
Callithrix jacchus	0.713864307	678	Primates	Mammal
Otolemur gernettii	0.728340675	681	Primates	Mammal
Galeopterus variegatus	0.722873900	682	Dermoptera	Mammal
Tupaia chinensis	0.730421687	664	Scandentia	Mammal
Mus musculus	0.745985401	685	Rodentia	Mammal
Rattus norvegicus	0.748538012	684	Rodentia	Mammal
Cricetulus griseus	0.740145985	685	Rodentia	Mammal
Cavia porcellus	0.740203193	689	Rodentia	Mammal
Octodon degus	0.733137830	682	Rodentia	Mammal
Chrysochloris asiatica	0.752186589	686	Afrosoricida	Mammal
Bos taurus	0.745985401	685	Artiodactyla	Mammal
Camelus ferus	0.742358079	687	Artiodactyla	Mammal
Sus scrofa	0.714701601	687	Artiodactyla	Mammal
Vicugna pacos	0.742358079	687	Artiodactyla	Mammal
Ailuropoda melanoleuca	0.698142415	646	Carnivora	Mammal
Canis lupus	0.740088106	681	Carnivora	Mammal
Leptonychotes weddelli	0.719530103	681	Carnivora	Mammal
Mustela putorius	0.741176471	680	Carnivora	Mammal
Odobenus rosmarus	0.725000000	680	Carnivora	Mammal
Orcinus orca	0.733624454	687	Cetacea	Mammal
Physeter catodon	0.736842105	684	Cetacea	Mammal
Erinaceus europaeus	0.750000000	704	Erinaceomorpha	Mammal
Ochotona princeps	0.734306569	685	Lagomorpha	Mammal
Elephantulus edwardii	0.746334311	682	Macroscelidea	Mammal
Pseudopodoces humilis	0.679104478	670	Passeriformes	Mammal
Ceratotherium simum	0.729809104	681	Perissodactyla	Mammal
Equus caballus	0.748538012	684	Perissodactyla	Mammal
Loxodonta africana	0.740524781	686	Proboscidea	Mammal
Trichechus manatus latirostris	0.740524781	686	Sirenia	Mammal
Eptesicus fuscus	0.743024963	681	Chiroptera	Mammal
Myotis brandtii	0.744493392	681	Chiroptera	Mammal
Pteropus alecto	0.746705710	683	Chiroptera	Mammal
Condylura cristata	0.696925329	683	Soricomorpha	Mammal

Table S5.1. Disorder proportion for FOXP4 (continued).

Species	Disorder Proportion	AA	Order	Class
Sorex araneus	0.751803752	693	Soricomorpha	Mammal
Orycteropus afer afer	0.740145985	685	Tubulidentata	Mammal
Serinus canaria	0.685543964	671	Passeriformes	Bird
Taeniopygia guttata	0.697604790	668	Passeriformes	Bird
Falco peregriunus	0.697014925	670	Falconiformes	Bird
Calypte anna	0.681470138	653	Trochiliformes	Bird
Aptenodytes forsteri	0.674772036	658	Sphenisciformes	Bird
Gallus gallus	0.689759036	664	Galliformes	Bird
Anas platyrhynchos	0.674174174	666	Anseriformes	Bird
Alligator mississipiensis	0.695522388	670	Crocodilia	Reptilia
Alligator sinensis	0.693693694	666	Crocodilia	Reptilia
Python bivittatus	0.678624813	669	Squamata	Reptilia
Anolis carolinensis	0.639344262	671	Squamata	Reptilia
Chelonia mydas	0.693693694	666	Testudines	Reptilia
Chrysemys picta bellii	0.695067265	669	Testudines	Reptilia
Pelodiscus sinensis	0.680303030	660	Testudines	Reptilia
Xenopus tropicalis	0.708527132	645	Anura	Amphibia
Xenopus laevis	0.705148206	641	Anura	Amphibia
