# Supplementary material for: FOXP in Tetrapoda: Intrinsically Disordered Regions, Short Linear Motifs and their evolutionary significance
Source: Genet Mol Biol. 2017 Mar 2;40(1):181–90. doi: 10.1590/1678-4685-GMB-2016-0115 (PMC5409772; doi:10.1590/1678-4685-GMB-2016-0115)
Supplement: Supplementary file 4 [file 1415-4757-gmb-1678-4685-GMB-2016-0115-Suppl04.pdf]

Table S3.1 Disorder proportion for FOXP2 orthologues.

Species	Disorder Proportion	AA	Order	Class
Homo sapiens	0.702097902	715	Primates	Mammal
Pan troglodytes	0.702513966	716	Primates	Mammal
Gorilla gorilla	0.701262272	713	Primates	Mammal
Pongo abelii	0.684431978	713	Primates	Mammal
Nomascus leucogenys	0.701262272	713	Primates	Mammal
Macaca mulatta	0.701680672	714	Primates	Mammal
Papio anubis	0.701680672	714	Primates	Mammal
Chlorocebus sabaeus	0.701680672	714	Primates	Mammal
Saimiri boliviensis boliviensis	0.701680672	714	Primates	Mammal
Callithrix jacchus	0.705469845	713	Primates	Mammal
Galeopterus variegatus	0.690834473	731	Dermoptera	Mammal
Tupaia chinensis	0.701680672	714	Scandentia	Mammal
Mus musculus	0.701680672	714	Rodentia	Mammal
Rattus norvegicus	0.701408450	710	Rodentia	Mammal
Cricetulus griseus	0.704323570	717	Rodentia	Mammal
Octodon degus	0.708913649	718	Rodentia	Mammal
Oryctolagus cuniculus	0.702513966	716	Lagomorpha	Mammal
Ochotona princeps	0.705382436	706	Lagomorpha	Mammal
Physeter catodon	0.689265537	708	Cetacea	Mammal
Vicugna pacos	0.704225352	710	Artiodactyla	Mammal
Camelus ferus	0.704225352	710	Artiodactyla	Mammal
Ceratotherium simum simum	0.701262272	713	Perissodactyla	Mammal
Felis catus	0.698727015	707	Carnivora	Mammal
Panthera tigris	0.689404934	689	Carnivora	Mammal
Odobenus rosmarus divergens	0.699576869	709	Carnivora	Mammal
Chrysochloris asiatica	0.701262272	713	Afrosoricida	Mammal
Echinops telfairi	0.711888111	715	Afrosoricida	Mammal
Elephantulus edwardii	0.700842697	712	Macroscelidea	Mammal
Eptesicus fuscus	0.704481793	714	Chiroptera	Mammal
Myotis brandtii	0.704067321	713	Chiroptera	Mammal
Pteropus alecto	0.704323570	717	Chiroptera	Mammal
Erinaceus europaeus	0.703389830	708	Erinaceomorpha	Mammal
Condylura cristata	0.701680672	714	Soricomorpha	Mammal
Orycteropus afer afer	0.698033707	712	Tubulidentata	Mammal
Loxodonta africana	0.697609001	711	Proboscidea	Mammal
Trichechus manatus latirostris	0.705801105	724	Sirenia	Mammal
Monodelphis domestica	0.700987306	709	Didelphimorphia	Mammal
Taeniopygia guttata	0.701828410	711	Passeriformes	Bird
Serinus canaria	0.702247191	712	Passeriformes	Bird
Manacus vitellinus	0.698727016	707	Passeriformes	Bird
Ficedula albicollis	0.712328767	730	Passeriformes	Bird
Melopsittacus undulatus	0.700987306	709	Psittaciformes	Bird

Table S3.1 Disorder proportion for FOXP2 orthologues (continued).

Species	Disorder Proportion	AA	Order	Class
Zonotrichia albicollis	0.700987306	709	Passeriformes	Bird
Falco peregrinus	0.715862069	725	Falconiformes	Bird
Aptenodytes forsteri	0.700564972	708	Sphenisciformes	Bird
Calypte anna	0.707015131	727	Trochiliformes	Bird
Gallus gallus	0.700564971	708	Galliformes	Bird
Anas platyrhynchos	0.702247191	712	Anseriformes	Bird
Python bivittatus	0.681818182	704	Squamata	Reptilia
Anolis carolinensis	0.710124827	721	Squamata	Reptilia
Pelodiscus sinensis	0.699290780	705	Testudines	Reptilia
Chelonia mydas	0.702549575	706	Testudines	Reptilia
Xenopus laevis	0.644475920	706	Anura	Amphibia
Xenopus tropicalis	0.616531165	738	Anura	Amphibia
Babina daunchina	0.656862745	714	Anura	Amphibia
Pachytriton labiatus	0.607142857	728	Caudata	Amphibia
