# Supplementary material for: FOXP in Tetrapoda: Intrinsically Disordered Regions, Short Linear Motifs and their evolutionary significance
Source: Genet Mol Biol. 2017 Mar 2;40(1):181–90. doi: 10.1590/1678-4685-GMB-2016-0115 (PMC5409772; doi:10.1590/1678-4685-GMB-2016-0115)
Supplement: Supplementary file 5 [file 1415-4757-gmb-1678-4685-GMB-2016-0115-Suppl10.pdf]

Table S6.1. Whole protein comparison for FOXP1 linear motifs content.

	**Linear Motifs**	CLV_C14_Caspase3-7	DEG_APCC_DBOX_1	DEG_SCF_FBW7_2	DOC_CKS1_1	DOC_CYCLIN_1	DOC_MAPK_1	DOC_PP2B_2	DOC_USP7_1	DOC_USP7_2	DOC_WW_Pin1_4	LIG_14-3-3_2	LIG_14-3-3_3	LIG_BRCT_BRCA1_1	LIG_CtBP_PxDLS_1	LIG_FHA_1	LIG_FHA_2	LIG_NRBOX	LIG_PCNA_PIPBox_1	LIG_PTAP_UEV_1	LIG_SUMO_SBM_1	LIG_SUMO_SBM_2	LIG_WD40_WDR5_1	MOD_CDK_1	MOD_CK1_1	MOD_CK2_1	MOD_GSK3_1	MOD_NEK2_1	MOD_NEK2_2	MOD_PIKK_1	MOD_PKA_1	MOD_PKA_2	MOD_PLK	MOD_ProDKin_1	MOD_SUMO	TRG_NES_CRM1_1	Total
**Mammals**	*Homo sapiens*		1	1	7	2	3	1	3		15	1	4	1	2	6	2	1	1	1	2		1	4	14	8	22	10	1	2	2	3	1	15	2	1	140
	Pan troglodytes		1	1	7	2	3	1	3		15	1	4	1	2	6	2	1	1	1	2		1	4	14	8	22	10	1	2	2	3	1	15	2	1	140
	Gorilla gorilla		1	1	7	2	3	1	3		15	1	4	1	2	6	2	1	1	1	2		1	4	14	8	22	10	1	2	2	3	1	15	2	1	140
	Pongo abelii		1	1	7	2	3	1	3		15	1	4	1	2	6	2	1	1	1	2		1	4	14	8	22	10	1	2	2	3	1	15	2	1	140
	Nomascus leucogenys		1	1	7	2	3	1	3		15	1	4	1	2	6	2	1	1	1	2		1	4	14	8	22	10	1	2	2	3	1	15	2	1	140
	Macaca mulatta		1	1	7	2	3	1	3		15	1	4	1	2	6	2	1	1	1	2		1	4	14	8	22	10	1	2	2	3	1	15	2	1	140
	Papio anubis		1	1	7	2	3	1	3		15	1	4	1	2	6	2	1	1	1	2		1	4	14	8	22	10	1	2	2	3	1	15	2	1	140
	Chlorocebus sabaeus		1	1	7	2	3	1	3		15	1	4	1	2	6	2	1	1	1	2		1	4	14	8	22	10	1	2	2	3	1	15	2	1	140
	Saimiri boliviensis		1	1	7	2	3	1	3		15	1	4	1	2	6	2	1	1	1	2		1	4	14	8	22	10	1	2	2	3	1	15	2	1	140
	Callithrix jacchus		1	1	7	2	3	1	3		15	1	4	1	2	6	2	1	1	1	2		1	4	14	8	22	10	1	2	2	3	1	15	2	1	140
	Galeopterus variegatus		1	1	7	2	3	1	3		15	1	4	1	2	6	2	1	1	1	2		1	4	14	9	23	10	1	2	2	3	1	15	2	1	142
	Tupaia chinensis		1	1	7	2	3	1	3		15	1	4	1	2	6	2	1	1	1	2		1	4	14	7	24	10	1	2	2	3	1	15	2	1	141
	Mus musculus		1	1	7	1	3	1	4		14	1	4	1	2	3	2	1	1	1	2			4	15	8	23	11	1	2	2	4	1	14	2	1	138
	Rattus norvegicus		1	1	7	1	3	1	3		13	1	4	1	2	4	3	1	1	1	2			3	15	8	22	10	1	2	2	4	1	13	2	1	134
	Cricetulus griseus		1	1	7	1	3	1	3		14	1	4	1	2	4	2	1	1	1	2			4	14	8	21	11	1	2	2	4	1	14	2	1	135
	Octodon degus		1	1	7	2	3	1	3		14	1	4	1	2	5	2	1	1	1	2			4	12	8	21	10	1	2	2	3	1	14	2	1	133
	Oryctolagus cuniculus		1	1	7	2	3	1	3		15	1	4	1	2	8	2	1	1	1	2		1	4	14	9	23	11	1	2	2	3	1	15	2	1	145
	Ochotona princeps		1	1	7	2	3	1	4		16	1	4	1	2	8	2	1	1	1	2		1	4	14	9	24	11	1	2	2	3	1	16	2	1	149
	Physeter catodon		1	1	7	2	3	1	3		15	1	4	1	2	5	2	1	1	1	2			4	14	7	25	10	1	2	2	3	1	15	2	1	140

Table S6.1. Whole protein comparison for FOXP1 linear motifs content (continued).

	Linear Motifs	CLV_C14_Caspase3-7	DEG_APCC_DBOX_1	DEG_SCF_FBW7_2	DOC_CKS1_1	DOC_CYCLIN_1	DOC_MAPK_1	DOC_PP2B_2	DOC_USP7_1	DOC_USP7_2	DOC_WW_Pin1_4	LIG_14-3-3_2	LIG_14-3-3_3	LIG_BRCT_BRCA1_1	LIG_CtBP_PxDLS_1	LIG_FHA_1	LIG_FHA_2	LIG_NRBOX	LIG_PCNA_PIPBox_1	LIG_PTAP_UEV_1	LIG_SUMO_SBM_1	LIG_SUMO_SBM_2	LIG_WD40_WDR5_1	MOD_CDK_1	MOD_CK1_1	MOD_CK2_1	MOD_GSK3_1	MOD_NEK2_1	MOD_NEK2_2	MOD_PIKK_1	MOD_PKA_1	MOD_PKA_2	MOD_PLK	MOD_ProDKin_1	MOD_SUMO	TRG_NES_CRM1_1	Total
**Mammals**	*Vicugna pacos*		1	1	7	2	3	1	3		16	1	4	1	2	6	2	1	1	1	2		1	4	14	8	22	10	1	3	2	3	1	16	2	1	143
	Odobenus rosmarus divergens		1		6	2	3	1	3		14	1	4	1	2	5	1	1	1	1	2	1		3	14	9	23	10	1	2	2	3	1	14	2	1	135
	Myotis brandtii	1	1	1	7	2	3	2	4		14	1	4	1	2	3	2	1	1	1	2		1	4	11	7	19	7	1	2	2	3	1	14	2	1	128
	Eptesicus fuscus	1	1	1	7	3	3	2	4		14	1	3	1	2	2	2	1	1	1	2		1	4	11	6	19	6	1	2	2	3	1	14	2	1	125
	Pteropus alecto		1	1	7	2	3	1	3		15	1	4	1	2	6	2	1	1	1	2		1	4	13	9	21	10	1	2	2	3	1	15	2	1	139
	Erinaceus europaeus		1	1	7	1	3	1	3		15	1	4	1	2	6	2	1	1	1	2		1	4	14	9	23	10	1	2	2	3	1	15	2	1	141
	Condylura cristata		1	1	7	2	3	1	3		15	1	4	1	2	6	2	1	1	1	2		1	4	12	9	23	11	1	2	2	3	1	15	2	1	141
	Echinops telfairi		1	1	8	1	3	1	3		14	1	4	1	2	6	2	1	1		2			3	12	7	19	10	1	2	2	3	2	14	2	1	130
	Chrysochloris asiatica		2	2	8	2	3	1	4		16	1	4	1	2	6	2	1	1		2		1	4	12	9	23	11		2	2	3	1	16	2	1	145
	Elephantulus edwardii		1	1	8	2	3	1	3		15	1	4	1	2	4	1	1	1		2		1	4	13	7	20	9	1	5	2	3	1	15	2	1	135
	Orycteropus afer afer		1	1	8	1	3	1	3		15	1	4	1	2	6	2	1	1		2		1	3	12	9	21	11	1	2	2	3	1	15	2	1	137
	Trichechus manatus latirostris		1	1	8	1	3	1	3		16	1	4	1	2	7	1	1	1		2	2	1	4	15	8	23	10	1	2	2	3	1	16	2	1	145
	Loxodonta africana		1	1	7	1	3	1	3		14	1	4	1	2	5	3	1	1		2	2	1	4	14	9	23	10	1	3	2	3	1	14	2	1	141
	Monodelphis domestica		1	1	9	2	3	1	3		18	1	4	1	2	4	2	1	1		2			4	12	6	20	9		2	2	3	1	18	2	1	136
**Birds**	*Serinus canaria*		1	1	8	1	3	1	3		16		4	1	2	7	3	1	1	1	2	2		4	12	8	20	10	1	2	2	3	2	16	2	1	141
	Zonotrichia albicollis		1	1	8	1	3	1	3	1	16		4	1	2	7	3	1	1	1	2	2		4	12	7	20	10	1	2	2	3	2	16	2	1	141
	Ficedula albicollis		1	1	8	1	3	1	3		16		4	1	2	7	3	1	1	1	2	2		4	12	8	20	10	1	2	2	3	2	16	2	1	141
	Taeniopygia guttata		1	1	8	2	3	1	3		17		4	1	2	7	3	1	1	1	2	2		4	13	8	21	10	1	2	2	3	2	17	2	1	146
	Manacus vitellinus		1	1	8	2	3	1	3		16		4	1	2	7	3	1	1	1	2	2		4	12	8	21	10	1	2	2	3	2	16	2	1	143
	Melopsittacus undulatus		1	1	8	1	3	1	3		16		3	1	2	7	3	1	1	1	2	2		4	12	8	20	10	1	2	2	3	2	16	2	1	140

Table S6.1. Whole protein comparison for FOXP1 linear motifs content (continued).

	**Linear Motifs**	CLV_C14_Caspase3-7	DEG_APCC_DBOX_1	DEG_SCF_FBW7_2	DOC_CKS1_1	DOC_CYCLIN_1	DOC_MAPK_1	DOC_PP2B_2	DOC_USP7_1	DOC_USP7_2	DOC_WW_Pin1_4	LIG_14-3-3_2	LIG_14-3-3_3	LIG_BRCT_BRCA1_1	LIG_CtBP_PxDLS_1	LIG_FHA_1	LIG_FHA_2	LIG_NRBOX	LIG_PCNA_PIPBox_1	LIG_PTAP_UEV_1	LIG_SUMO_SBM_1	LIG_SUMO_SBM_2	LIG_WD40_WDR5_1	MOD_CDK_1	MOD_CK1_1	MOD_CK2_1	MOD_GSK3_1	MOD_NEK2_1	MOD_NEK2_2	MOD_PIKK_1	MOD_PKA_1	MOD_PKA_2	MOD_PLK	MOD_ProDKin_1	MOD_SUMO	TRG_NES_CRM1_1	Total
**Birds**	*Falco peregrinus*		1	1	8	1	3	1	3		16		4	1	2	7	3	1	1	1	2	2		4	12	8	20	10	1	2	2	3	2	16	2	1	141
	Aptenodytes forsteri		1	1	8	1	3	1	3		16		4	1	2	8	3	1	1	1	2	2		4	12	8	20	10	1	2	2	3	2	16	2	1	142
	Calypte anna		1	1	8	1	3	1	3		15		4	1	2	7	3	1	1	1	2	2		3	12	7	19	10	1	2	2	3	2	15	2	1	136
	Anas platyrhynchos		1	1	8	2	3	1	3		16		4	1	2	7	3	1	1	1	2	2		4	12	8	21	10	1	3	2	3	2	16	2	1	144
	Gallus gallus		1	1	8	1	3	1	3		16		4	1	2	7	3	1	1	1	2	2		4	12	8	20	10	1	2	2	3	2	16	2	1	141
	Python bivittatus		1	2	7	1	3	1	4		14		3	1	2	4	4	1	1	1	2	1		3	12	7	21	9		2	2	3	2	14	2	1	131
	Anolis carolinensis		1	1	7	1	3	1	3		15	1	4	1	2	6	3	1	1	1	2	2		3	14	9	23	11	1	2	2	3	2	15	2	1	144
	Chelonia mydas	1	1	1	9	1	3	1	3		17		5	1	2	7	3	1	1		2	2		4	12	7	21	10	1	1	1	2	3	17	2	1	143
	Pelodiscus sinensis	1	1	1	9	1	3	1	3		17		4	1	2	7	3	1	1		2	2		4	12	7	21	10	1	1	1	2	3	17	2	1	142
**Amphibian**	*Xenopus laevis*	1	2		6	1	1		6		13		3	2	2	8	3	1	1	1	2	3		2	10	5	17	9		3	2	3	1	13	2	1	124
