# Supplementary material for: FOXP in Tetrapoda: Intrinsically Disordered Regions, Short Linear Motifs and their evolutionary significance
Source: Genet Mol Biol. 2017 Mar 2;40(1):181–90. doi: 10.1590/1678-4685-GMB-2016-0115 (PMC5409772; doi:10.1590/1678-4685-GMB-2016-0115)
Supplement: Supplementary file 6 [file 1415-4757-gmb-1678-4685-GMB-2016-0115-Suppl13.pdf]

Table S6.4. Whole protein comparison for FOXP4 linear motifs content.

	**Linear Motifs**	CLV_C14_Caspase3-7	DEG_APCC_DBOX_1	DEG_ODPH_VHL_1	DEG_SCF_FBW7_1	DEG_SIAH_1	DOC_ANK_TNKS_1	DOC_CKS1_1	DOC_CYCLIN_1	DOC_MAPK_1	DOC_PIKK_1	DOC_PP1_RVXF_1	DOC_PP2B_2	DOC_USP7_1	DOC_USP7_2	DOC_WW_Pin1_4	LIG_14-3-3_2	LIG_14-3-3_3	LIG_BRCT_BRCA1_1	LIG_BRCT_BRCA1_2	LIG_CtBP_PxDLS_1	LIG_FHA_1	LIG_FHA_2	LIG_MAD2	LIG_MYND_1	LIG_NRBOX	LIG_SUMO_SBM_1	LIG_SUMO_SBM_2	MOD_CDK_1	MOD_CK1_1	MOD_CK2_1	MOD_GSK3_1	MOD_NEK2_1	MOD_NEK2_2	MOD_PIKK_1	MOD_PKA_1	MOD_PKA_2	MOD_PKB_1	MOD_PLK	MOD_ProDKin_1	MOD_SUMO	TRG_NES_CRM1_1	TRG_NLS_MonoExtC_3	Total
**Mammals**	*Homo sapiens*	4	1			1		3	2	1		1	4	9		14	1		2	1			3		1	1	2	2	3	10	8	14	5	3	2	4	6		2	14	2		1	127
	Pan paniscus	4	1			1		3	2	1		1	4	8		14	1		2	1			3		1	1	2	2	3	10	8	16	5	2	2	4	6		1	14	2		1	126
	Gorilla gorilla	4	1			1		3	2	1		1	4	10		14	1		2	1			3		1	1	2	2	3	11	8	14	5	3	2	4	6		2	14	2		1	129
	Pongo abelii	4	1		2	1		4	2	1		1	3	9		15	1		2	1			3		1	1	2	2	3	10	8	15	5	3	2	4	6		2	15	2		1	132
	Nomascus leucogenys	4	1			1		3	2	1		1	4	9		14	1		2	1			3		1	1	2	2	3	10	8	15	5	3	2	4	6		2	14	2		1	128
	Macaca mulatta	4	1		2	1		4	2	1		1	3	8		15	1		2	1			3		1	1	2	2	3	11	7	16	5	3	2	4	6		2	15	2		1	132
	Papio anubis	4	1		2	1		4	2	1		1	3	8		15	1		2	1			3		1	1	2	2	3	11	7	16	5	3	2	4	6		2	15	2		1	132
	Chlorocebus sabaeus	4	1		2	1		4	2	1		1	3	7		15	1		2	1			3		1	1	2	2	3	11	7	16	5	3	2	4	6		2	15	2		1	131
	Saimiri boliviensis	4	1			1		3	2	1		1	4	10		14	1		2	1			3		1	1	2	2	3	12	7	15	5	3	3	4	5		2	14	2		1	130
	Callithrix jacchus	4	1			1		3	2	1		1	3	9		13	1		1				3		1	1	2	2	3	12	7	16	6	3	3	4	6		2	13	2		1	127
	Otolemur garnettii	4	1			1		3	2	1		1	5	8		14	1		2	1		1	3		2	1	2	2	3	12	7	12	5	2	2	4	6		2	14	2		1	127
	Galeopterus variegatus	4	1			1		3	2	1		1	4	9		14	1		2	1			3		2	1	2	2	3	16	7	16	5	2	3	4	6		2	14	2	1	1	136
	Tupaia chinensis	2	1			1		3	2	1		1	5	5		15	1		1				2		1	1	2	2	3	9	7	11	5	3	2	4	6		2	15	2		1	116
	Mus musculus	1	1			1		3	2	1		1	5	7		13	1		1	1			1		1	1	2	2	4	12	8	13	4	2	5	3	5		1	13	1		1	117
	Rattus norvegicus	2	1			1		3	2	1		1	4	6		15	1		1	1			1		1	1	2	2	4	11	8	14	3	2	5	3	5		1	15	1		1	119

Table S6.4. Whole protein comparison for FOXP4 linear motifs content (continued).

	**Linear Motifs**	CLV_C14_Caspase3-7	DEG_APCC_DBOX_1	DEG_ODPH_VHL_1	DEG_SCF_FBW7_1	DEG_SIAH_1	DOC_ANK_TNKS_1	DOC_CKS1_1	DOC_CYCLIN_1	DOC_MAPK_1	DOC_PIKK_1	DOC_PP1_RVXF_1	DOC_PP2B_2	DOC_USP7_1	DOC_USP7_2	DOC_WW_Pin1_4	LIG_14-3-3_2	LIG_14-3-3_3	LIG_BRCT_BRCA1_1	LIG_BRCT_BRCA1_2	LIG_CtBP_PxDLS_1	LIG_FHA_1	LIG_FHA_2	LIG_MAD2	LIG_MYND_1	LIG_NRBOX	LIG_SUMO_SBM_1	LIG_SUMO_SBM_2	MOD_CDK_1	MOD_CK1_1	MOD_CK2_1	MOD_GSK3_1	MOD_NEK2_1	MOD_NEK2_2	MOD_PIKK_1	MOD_PKA_1	MOD_PKA_2	MOD_PKB_1	MOD_PLK	MOD_ProDKin_1	MOD_SUMO	TRG_NES_CRM1_1	TRG_NLS_MonoExtC_3	Total
**Mammals**	*Cricetulus griseus*	2	1			1		3	3	2		1	4	6		13	1		2	1		1	1		1	1	2	2	4	12	7	15	4	3	4	4	7		2	13	1		1	125
	Octodon degus	1	1			1		4	2	2		1	3	6		15	1	1	1			1	1		1	1	2	5	4	9	7	15	6	3	4	4	6		2	15	2		1	128
	Cavia porcellus	1	1					3	2	1		1	3	8		14	1	1	1				1		1	1	2	2	3	12	7	13	5	3	4	4	6		2	14	2		1	120
	Ochotona princeps	3	1			1	1	3	2	1		1	4	7		15	1	1	2	1			2		2	1	2	2	3	13	7	14	5	3	3	4	6		2	15	2		1	131
	Physeter catodon	2	1					4	2	1		1	4	9		15	1		2	1			1		2	1	2	2	4	12	7	12	5	3	3	3	5		2	15	2		1	125
	Orcinus orca	2	1			1		4	2	1		1	5	9		16	1		2	1			1		2	1	2	2	4	12	7	11	5	3	2	4	6		2	16	2		1	129
	Bos taurus	2	1			1		4	2	1		1	3	7		15	1		2	1			1		1	1	1	2	4	13	7	13	5	3	3	4	7		2	15	2		1	126
	Sus scrofa	2	1			1	1	4	2	1		1	4	7		15	1	1	2	1	1		1		2	1	2	2	4	12	7	11	5	3	2	4	7		2	15	2		1	128
	Camelus ferus	2	1			1		4	2	1		1	4	8		15	1		2	1		1	1		2	1	2	5	4	14	7	13	4	3	2	4	6		2	15	2		1	132
	Vicugna pacos	2	1			1		4	2	1		1	4	8		15	1		2	1		1	1		2	1	2	5	4	14	7	13	4	3	2	4	6		2	15	2		1	132
	Ceratotherium simum simum	2	1			1	1	4	2	1		1	4	8		15	1		2	1			1		1	1	2	2	4	13	7	13	5	2	3	4	7		2	15	2		1	129
	Equus caballus	3	1			1		4	2	1		1	4	9		15	2		2	1			2		2	1	2	2	4	12	7	14	5	2	2	4	6		2	15	2		1	131
	Leptonychotes weddellii	3	1			1		4	2	1		1	3	7		15	1		1				2		2	1	2	1	4	14	8	12	5	2	3	4	7		2	15	2		1	127
	Odobenus rosmarus divergens	3	1			1		4	2	1		1	4	8		15	1		2	1			2		2	1	2	1	4	15	8	13	5	2	3	4	7		2	15	2		1	133
	Mustela putorius furo	2	1			1		4	2	1		1	4	8		15	1		2	1			1		2	1	2	2	4	15	7	11	5	2	1	4	7		2	15	2		1	127

Table S6.4. Whole protein comparison for FOXP4 linear motifs content (continued).

	Linear Motifs	CLV_C14_Caspase3-7	DEG_APCC_DBOX_1	DEG_ODPH_VHL_1	DEG_SCF_FBW7_1	DEG_SIAH_1	DOC_ANK_TNKS_1	DOC_CKS1_1	DOC_CYCLIN_1	DOC_MAPK_1	DOC_PIKK_1	DOC_PP1_RVXF_1	DOC_PP2B_2	DOC_USP7_1	DOC_USP7_2	DOC_WW_Pin1_4	LIG_14-3-3_2	LIG_14-3-3_3	LIG_BRCT_BRCA1_1	LIG_BRCT_BRCA1_2	LIG_CtBP_PxDLS_1	LIG_FHA_1	LIG_FHA_2	LIG_MAD2	LIG_MYND_1	LIG_NRBOX	LIG_SUMO_SBM_1	LIG_SUMO_SBM_2	MOD_CDK_1	MOD_CK1_1	MOD_CK2_1	MOD_GSK3_1	MOD_NEK2_1	MOD_NEK2_2	MOD_PIKK_1	MOD_PKA_1	MOD_PKA_2	MOD_PKB_1	MOD_PLK	MOD_ProDKin_1	MOD_SUMO	TRG_NES_CRM1_1	TRG_NLS_MonoExtC_3	Total
**Mammals**	*Ailuropoda melanoleuca*	2	1			1		3	2	1		1	3	8		14	1		2	1			1		2	1	2	2	4	12	7	13	5	2	4	4	6		2	14	2		1	124
	Canis lupus familiaris	2	1			1		4	3	1		1	3	7		15	1		2	1			1		2	1	2	2	4	14	8	12	5	2	4	4	6		2	15	2		1	129
	Myotis brandtii	2	1			1		4	2	1		1	4	7		15	1		2	1			1		2	1	2	2	4	13	6	13	5	3	3	4	6		2	15	2		1	127
	Eptesicus fuscus	2	1			1		4	2	1		1	4	6		15	1		2	1			1		2	1	2	2	4	13	6	13	5	3	3	4	6		2	15	2		1	126
	Pteropus alecto	2	1			1		4	2	1		1	4	7		15	1		2	1			1		2	1	2	2	4	11	6	11	5	3	2	4	7		2	15	2		1	123
	Erinaceus europaeus	2	1			1		4	2	2		1	3	10		14	1	1				2	2		1	1	3	6	3	17	7	21	5	2	4	3	7		2	14	2		1	145
	Sorex araneus	2	1			1		4	2	1		1	4	11		15	1						1		1	1	1	1	2	12	7	12	5	3	2	3	5		2	15	2		1	119
	Condylura cristata	2	1			1		4	2	1		1	4	7		14	1		1				1		2	1	2	2	4	11	7	10	6	2	3	4	7		1	14	2		1	119
	Chrysochloris asiatica	2	1			1		4	2	1		1	4	5	1	16	1		2	1		2	1		3	1	3	4	4	11	8	13	6	3	1	4	6	1	2	16	2		1	134
	Elephantulus edwardii	2	1			1		4	2	1		1	4	6		16	1		1	1		1	2		2	1	2	4	4	8	7	10	5	3	2	4	6		2	16	2		1	123
	Orycteropus afer afer	2	1			1		4	2	1		1	5	7		15	1		2	1		2	1		2	1	2	4	4	11	7	13	5	3	3	4	6	1	2	15	2		1	132
	Trichechus manatus latirostris	2	1			1		4	2	1		1	4	8		16	1		2	1		2	1		2	1	2	4	4	12	7	12	5	3	3	4	7	1	2	16	2		1	135
	Loxodonta africana	3	1			1		4	2	1		1	4	9		16	1		2	1		2	2		2	1	2	4	4	12	7	20	5	3	4	4	5		2	16	2		1	144
**Birds**	*Serinus canaria*	1	1					5	2	1	2	1	1	2		16	1	1	3		1	3	3		1	1	2		3	11	8	26	9	2	3	3	5		3	16	2		1	140
	Pseudopodoces humilis	1	1					5	2	1	2	1	1	2		16	1	1	3		1	2	3		1	1	2		3	11	8	25	10	2	2	3	5		3	16	2		1	138

Table S6.4. Whole protein comparison for FOXP4 linear motifs content (continued).

	Linear Motifs	CLV_C14_Caspase3-7	DEG_APCC_DBOX_1	DEG_ODPH_VHL_1	DEG_SCF_FBW7_1	DEG_SIAH_1	DOC_ANK_TNKS_1	DOC_CKS1_1	DOC_CYCLIN_1	DOC_MAPK_1	DOC_PIKK_1	DOC_PP1_RVXF_1	DOC_PP2B_2	DOC_USP7_1	DOC_USP7_2	DOC_WW_Pin1_4	LIG_14-3-3_2	LIG_14-3-3_3	LIG_BRCT_BRCA1_1	LIG_BRCT_BRCA1_2	LIG_CtBP_PxDLS_1	LIG_FHA_1	LIG_FHA_2	LIG_MAD2	LIG_MYND_1	LIG_NRBOX	LIG_SUMO_SBM_1	LIG_SUMO_SBM_2	MOD_CDK_1	MOD_CK1_1	MOD_CK2_1	MOD_GSK3_1	MOD_NEK2_1	MOD_NEK2_2	MOD_PIKK_1	MOD_PKA_1	MOD_PKA_2	MOD_PKB_1	MOD_PLK	MOD_ProDKin_1	MOD_SUMO	TRG_NES_CRM1_1	TRG_NLS_MonoExtC_3	Total
**Birds**	*Taeniopygia guttata*	1	1					5	2	1	2	1	1	3		15	1	1	3		1	1	3		1	1	2	1	3	11	8	22	8	2	2	4	6		3	15	2	1	1	135
	Falco peregrinus	1	1					5	2	1	2	1	1	2		16	1	1	3		1	3	4		1	1	2		3	12	9	25	9	2	4	3	5		3	16	2		1	143
	Aptenodytes forsteri	1	1					5	2	1	2	1	1	3		16	1	1	3		1	2	4		1	1	2		3	12	9	25	9	2	3	3	5		3	16	2		1	142
	Calypte anna	1	1			1		5	2	1	2	1	1	3		16	1	2	3		1	2	4		1	1	2		3	12	9	23	8	2	3	3	6		3	16	2		1	142
	Anas platyrhynchos	1	1	1				5	4	1	2	1	2	3		15	1	1	4		1	2	3		2	1	2		3	12	7	24	8	2	2	3	5		2	15	2	1	1	140
	Gallus gallus	1	1					5	2	1	2	1	1	2		15	1	1	3		1	3	3		1	1	2		3	12	9	26	8	2	4	3	5		3	15	2		1	140
**Reptilia**	*Alligator mississippiensis*	1	1					5	2	1	2	1	1	1		15	1	1	3		1	3	4		1	1	2		3	11	9	24	9	2	4	3	5		2	15	2		1	137
	Alligator sinensis	1	1					5	2	1	2	1	1	1		15	1	1	3		1	3	4		1	1	2		3	11	9	24	9	2	4	3	5		2	15	2		1	137
	Python bivittatus	1	1					4	2	1	2	1	1	1		15	1	1	3		1	3	5		1	1	2		2	12	9	25	9	1	4	3	5		2	15	2		1	137
	Anolis carolinensis	1	1					3	2	1	2	1	1	1		15	1		2		1	3	4		1	1	2		2	12	8	23	9	1	3	3	5		1	15	2		1	128
	Chrysemys picta bellii	1	1					4	2	1	2	1	1	1		14		1	2		1	3	4		1	1	2		3	13	9	25	9	1	4	2	4		2	14	2		1	132
	Chelonia mydas	1	1					4	2	1	2	1	1	1		14		1	2		1	3	4		1	1	2		3	12	9	25	10	1	4	2	4		2	14	2		1	132
	Pelodiscus sinensis	1	1					4	2	1		1	1	1		14		1	2		1	4	3		1	1	2		3	12	8	23	10	1	4	2	4		2	14	2		1	127
**Amphibia**	*Xenopus laevis*	1	1					4	4	1		1		5		15	2				1	5	4	1	1	1	2		2	15	9	23	6	1	5	2	4		1	15	1		1	134
	Xenopus tropicalis	3	1					5	3	1		1	1	7		16	1				1	5	5	1	1	1	2		2	16	8	26	7	2	5	2	4		2	16	1		1	147
