# Supplementary material for: FOXP in Tetrapoda: Intrinsically Disordered Regions, Short Linear Motifs and their evolutionary significance
Source: Genet Mol Biol. 2017 Mar 2;40(1):181–90. doi: 10.1590/1678-4685-GMB-2016-0115 (PMC5409772; doi:10.1590/1678-4685-GMB-2016-0115)
Supplement: Supplementary file 7 [file 1415-4757-gmb-1678-4685-GMB-2016-0115-Suppl16.pdf]

Table S9. Eukaryotic linear motifs and their functions.

ELM	Evidence Class	Logical	Reability	Code	Probability	ELM Description	Functional Description
CLV_C14_Caspase3-7	Experimental	TP	Likely	a	0.0030937	The amino acids around the caspase-3 and -7 cleavage site are named N- to C-terminal: P4, P3, P3, P2, P1, P-1. The scissile bond between P1 and P-1 is cleaved by caspase-3 and -7, whereas positions P4 to P-1 are important for substrate specificity and recognition. P1 is always an aspartate (D), while P-1 is usually a small amino acid. Proline (P) as secondary alpha-amino acid is not accepted at P-1. An in vitro kinetic study argues for small amino acids, phenylalanine (F) or tyrosine and no ionic amino acids at P-1 (Stennicke,2000). The regular expression allows small amino acids at P-1. Other residues are still described but data was not valid enough to create an additional regular expression. The backbone of amino acids at P2 and P3 is stabilised by hydrogen (H) bonds allowing caspase-3 and -7 a broad spectrum of amino acids at these positions. At P2 non-polar amino acids (valine (V), leucine (L), P) are preferred because of possible interactions with a hydrophobic pocket. Threonine (T) is also very common. At P3 glutamate (E) is preferred because of an additional H-bond. However other amino acids like serine (S) or L are still common. In the regular expression P3 is not specified, except for the prohibition of P, because caspase-3 and -7 accept a variety of amino acids at P3. D is strongly preferred at P4 due to strong H-bond interactions, followed by S, T, and E. Crystal structures with pentapeptides argue for a preference for hydrophobic residues at P5 because of hydrophobic interactions with two F residues in case of caspase-3. This site is missing in caspase-7 (Fu,2008). The regular expression does not include P5 because caspase-3 cleaves also substrates with non-hydrophobic residues at P5. Nevertheless a hydrophobic residue at P5 is a hint that the protein is rather a caspase-3 substrate then a caspase-7 one. Based on the observed variations at P4-P2, the regular expression will on the one hand produce false positives and on the other hand not match all described cleavage sites.	The proteases caspases-3 and -7 play an important role in programmed cell death (apoptosis). Cleavage of the caspase substrates results in characteristic morphological features of apoptotic cell death, including membrane blebbing, pyknotic nuclei, cell rounding, and formation of apoptotic vesicles. Caspases recognise their substrates by a cleavage motif. The amino acids of the substrate around the caspase cleavage site are named N- to C-terminal: P4, P3, P2, P1, P-1. The scissile bond between the essential aspartate at P1 and P-1, usually a small amino acid, is cleaved by caspase-3 and -7, whereas positions P4 to P-1 are important for substrate specificity and recognition.
DEG_SCF_FBW7_1	Experimental	TP	Certain	b	0.0007138	FBW7 (also called FBXW7, hCdc4 or hSel10) is a member of a family of F-box proteins that binds via WD40 beta propeller to its substrates after their phospho-degron motifs (also named CPDs, i.e. Cdc4 phospho-degrons) have been doubly phosphorylated (Hao,2007, Welcker,2008). The core of the motif is TPxxS, preceded by a variable number of hydrophobic residues. The motif is used in cell cycle regulation: the widely conserved G2 phase-specific cyclin E destruction by FBW7 was first described in yeast. The Thr is often phosphorylated by GSK3, after priming at the other P-site, linking the FBW7 activity with the mitogenic signalling pathway. In some instances the Thr may alternatively be targeted by other kinases such as CDKs. Interestingly, many of the known FBW7 substrates are proto-oncogenes with key roles in the regulation of cell division, differentiation and growth. However, a proposed FBW7 phosphodegron in the key cell state monitor mTor (Mao,2008) does not match the diphosphorylated motif. The phosphodegron in v-Jun is mutated and inactivated, enhancing oncogenicity by preventing its destruction. Some variant motifs substitute a Glu residue for the second phosphosite, e.g. in SV40 large T, and this variant is represented by the alternative pattern in ELM.	Several phosphodegrons are required for cell state-dependent recognition of regulatory proteins by SCF complexes via repeat domains of associated F box proteins (FBPs) and their subsequent ubiquitin-mediated degradation. The SCF-FBW7 and the SCF-betaTrCP1 motifs, contain two phosphorylated residues, which are recognised via a WD40 domain. For example, the SCF-FBW7 degron TPxxS is found in cyclin E, which is required for the G1/S transition. The SCF-betaTrCP1 degron DSGxxS operates in a broader range of cell regulation. For example, NF-kappa-B inhibitors are phosphorylated and destroyed under immune stimulation while beta-catenin is degraded in the absence of Wnt signalling. Skp2, another FBP, recognises cell cycle regulators via its leucine-rich repeat. In case of the single-phosphorylated DEG_SCF_SKP2-CKS1_1 motif, Skp2 requires additional binding of Cks1 for recognition. So far, only a few cell cycle inhibitors, including p27Kip1 that is mainly involved in G1 arrest, have been found to carry this degron.
DOC_PP2B_2	Experimental	TP	Certain	c	0.0022964	This PP2B-docking motif is defined by four amino acids. There is an absolute requirement for leucine in the first position of the motif and for proline in the last position. However, the viral protein A238L (O36972), which has been shown to prevent recognition of LxvP-containing substrates by calcineurin, contains a lysine residue instead of a proline in the last position (4F0Z) (Grigoriu,2013). The second position can accommodate a variety of amino acids. The third position generally contains a valine, but other hydrophobic residues have also been observed. Some motif instances are immediately preceded by an aromatic residue, which can further strengthen the interaction. Upon binding of Ca2+, the CNA subunit undergoes a conformational change, exposing the hydrophobic motif-binding pocket that is located at the interface of the CNA and CNB subunits. Therefore, LxvP sites can only interact with activated calcineurin. The hydrophobic pocket includes two CNA residues (W352, F356) and three CNB residues (L115, M118, V119), which mediate binding to substrates containing an LxvP motif. Immunosuppressants have been shown to bind to the hydrophobic pocket in a similar way. Upon binding to the hydrophobic pocket, the motif adopts a conformation in which it is almost parallel to the α-helix of CNA binding to CNB. The proline in the motif is predicted to interact with the aromatic residues in CNA.	Calcineurin (PP2B) is a Ca2+- and calmodulin-regulated serine/threonine protein phosphatase known to affect cell biological function mainly in yeast and mammalian systems but is found in all Eukaryotes. It regulates a number of different pathways, including activation of the NFAT family of transcription factors, regulation of axonal guidance, the Ca2+-dependent migration of neutrophils, synaptic plasticity, and apoptosis. The effectiveness of dephosphorylation depends not only on the proximal position of calcineurin-binding site to the dephosphorylation site but also on the flexibility of the calcineurin active site. Calcineurin has been shown to interact with two distinct linear motifs: one docking motif (DOC_PP2B_1) that directly interacts with the catalytic subunit A (CNA) of calcineurin, and a second docking motif (DOC_PP2B_2) that binds to both CNA and calcineurin B (CNB), the regulatory Ca2+-binding subunit of calcineurin.
DOC_USP7_1	Experimental	TP	Certain	d	0.0123885	Targeting motif found in USP7 substrates, docking to the NTD domain. The USP7 N-terminal domain is a TRAF-like domain but with different sequence specificity to the classical TRAF domain. The motif identified in p53 and MDM2 recognizes the same surface groove in USP7. But MDM2s make more extensive contacts than p53, leading to stronger affinity. The motif identified in these proteins can therefore be categorized as either a lower affinity motif or high affinity motif, depending on the extent of contact. The general pattern of the motif is a simple P..S and will have frequent matches in cellular proteins but it is not clear how numerous are the USP7 substrates. CAUTION: This motif has poor discrimination and is likely to be poorly predictive.	USP7, also known as HAUSP, is a deubiquitinating enzyme which cleaves ubiquitin moieties from its substrates. The USP7-mediated deubiquitination of P53, Mdm2 and USP7 inhibition by the herpesviral proteins EBNA1 and ICP0 shows its importance in the regulation of cell survival pathways and controlling key cellular processes important for viral infection. The N-terminal TRAF-like domain of USP7 is responsible for substrate recognition and nuclear localization while the catalytic core domain is required for the deubiquitinating activity.

Table S9. Eukaryotic linear motifs and their functions (continued).

ELM	Evidence Class	Logical	Reability	Code	Probability	ELM Description	Functional Description
DOC_WW_Pin1_4	Experimental	TP	Certain	e	0.0154332	Proline-directed phosphorylation is a major cellular signalling mechanism. Proline directed kinases include Map kinases and cyclin-dependent kinases. Many p(S/T)P sites act as binding motifs for recruiting proteins or placing enzymes close to their substrates. Proline isomerisation may confound the function of these sites but also provides a mechanism for their regulation. This requires a peptidyl-prolyl cis/trans isomerase (PPIase). The phospho-specific PPIase is Pin1 (for Protein interacting with NIMA 1). It belongs to the parvulin subfamily of PPIases. Pin1 homologs are highly conserved in eukaryotes. Pin1 regulates diverse cellular process, including growth-signal response, cell-cycle progression, cellular stress response, neuronal function and immune response. Pin1 is an 18 kDa protein with two domains: the N-terminal WW domain and the C-terminal PPIase domain connected by a flexible linker. The WW domain acts as the binding module to bind its substrate p(S/T)P site and bring the catalytic PPIase domain close to its substrate where it catalyses cis-trans conversions. The Pin1 WW domain interaction with substrates occurs through a conserved phosphate-binding pocket, present in Pin1 and it homologues. The pSer/Thr-Pro peptides all bind to the WW domain in the trans conformation. Only WW domains which have an arginine in loop1 will be able to bind the sequence with core motif p(S/T)P. The phosphorylated residue is recognized by the Arginine as well as by backbone interactions. The Pro residue enters a pocket bounded by the second Tryptophan.	WW Domains are small but abundant domains found in diverse regulatory situations. The binding peptide motifs appear always to involve proline residues. Specific motifs vary for different WW domains and in some cases must be phosphorylated on a serine or threonine.
Lig_14-3-3_2	Experimental	TP	Certain	f	0.0015569	The longer mode 2 interacting phospho-motif for 14-3-3 proteins with key conservation RxxxS#p where # is a conserved hydrophobic position and p is a semiconserved Pro. Pro is excluded -1 and +1 of the pSer due to tight backbone interactions. Pro is usually depicted as required at +2 but (unlike mode 1) it is neither strongly conserved nor making tight contact to 14-3-3. Instead the +1 residue is always a conserved hydrophobic with an extensive packing face. Other residue preferences in the x positions are likely to affect binding affinity and some combinations may be disfavoured. The typical kinases phosphorylating mode 2 peptides are likely to have a positive charge preference preceding the phosphorylated residue.	14-3-3 proteins interact with specific phosphoserine or phosphothreonine containing motifs.
Lig_14-3-3_3	Experimental	TP	Certain	g	0.004948	Consensus derived from reported natural interactors which do not match the Mode 1 and Mode 2 ligands. Key conserved residues are missing. While the sequence range of 14-3-3 binders is certainly not fully defined, a pattern derived from outliers as here may be poorly predictive and matches should be treated with CAUTION. Validation is paramount.	14-3-3 proteins interact with specific phosphoserine or phosphothreonine containing motifs.
LIG_BRCT_BRCA1_1	Experimental	TP	Certain	h	0.0019115	The LIG_BRCT_BRCA1_1 motif binds with low affinity to the BRCT domain of BRCA1. The motif has the consensus sequence S..F and these residues are specially recognized by the binding pocket in the BRCT domains. The high affinity motif has an additional bound lysine residue (S..F.K).	BRCT domains are protein modules mainly found in Eukaryota. BRCT domains are present in proteins that are associated with DNA damage response. They recognize and bind specific phosphorylated serine (pS) sequences. This phospho-protein mediated interaction of the BRCT domain has a central role in cell-cycle check point and DNA repair functions.
LIG_CtBP_PxDLS_1	Experimental	TP	Likely	i	0.0001173	The PxDLS motif pattern is based on the conservation of reported sequence instances together with the structure of the CtBP domain in complex with a PxDLS peptide (1HL3). Beta-augmentation at the sheet edge places the peptide sidechains in specific places on the CtBP surface. Pro at position 1 makes an H-bond to strand edge backbone and fits in a hydrophobic pocket that will not accommodate other residues (Gly with no side chain may be the least disruptive). Position 2 contributes to beta augmentation so that the semi-conserved sidechain is placed in a shallow hydrophobic pocket, which also allows Glu due to proximity of surface positive charge. Position 3 is most often Asp, probably due to favourable charged residue proximity but is surface accessible and accepts some changes. Position 4 contributes to beta augmentation so that the sidechain enters a deep hydrophobic groove that fits to Leu and would probably allow Met but reject most other residues. Position 5 has a Ser-Thr preference but appears to accept R (as in HDACs) and some other mostly small residues. Following the core peptide there are clear preferences for Lys or Arg but these are not a strict requirement. However the conserved GLDLSKK motif in Hic1 is reported to bind CtBP but lacks Pro: Therefore for Gly at position 1 which must weaken the interaction, the motif in ELM requires C-terminal positive charge compensation.	The PxDLS motif is present in a number of nuclear proteins, including certain transcription factors and HDACs, that recruit CtBP (C-terminal binding protein) into nuclear complexes. CtBP has a generally repressive effect on transcription and must be removed to active CtBP regulated genes.
LIG_FHA_1	Experimental	TP	Certain	j	0.0086622	LIG_FHA_1 motifs are short phosphothreonine modules binding FHA domains with large aliphatic amino acids at the pT+3 position. The motif has the consensus sequence of T..[IVL]. Proteins with FHA domains having this preference include the checkpoint kinase chk2 (Li,2002) and DNA repair protein rad9 (Byeon,2001).	The FHA domain is a signal transduction module which recognizes phosphothreonine containing peptides on the ligand proteins. FHA domains partake in many signalling processes but are especially prevalent in nuclear proteins that are involved in cell cycle checkpoint, DNA repair and transcriptional regulation.
LIG_FHA_2	Experimental	TP	Certain	k	0.0082864	LIG_FHA_2 motifs are short phosphothreonine peptide modules contains acidic amino acids at the pT+3 position. The motif has the consensus sequence of T..[ED]. FHA domains with this preference are found in checkpoint/repair proteins MRC1 and Rad9 of fungi and metazoan Xrcc1 (Luo,2004) and Xrcc4 (Koch,2004).	The FHA domain is a signal transduction module which recognizes phosphothreonine containing peptides on the ligand proteins. FHA domains partake in many signalling processes but are especially prevalent in nuclear proteins that are involved in cell cycle checkpoint, DNA repair and transcriptional regulation.
LIG_MYND_1	Experimental	TP	Certain	l	0.0006499	The PxLxP motif is recognized by a subset of MYND domain containing proteins. The target specificity is mainly due to the highly charged surface of the MYND domain with a positive face consisting of C-terminal residues on one side, and a negatively charged region on the other side. The positive charge of these side chains could mediate long-range electrostatic interactions with the negative charges that may flank the PxLxP motif. So the interaction might require a longer region, while the PxLxP motif constitutes the core motif.	The MYND domain is a zinc binding domain that is involved in protein-protein interactions mainly in the context of transcriptional regulation. It is named after Myeloid, Nervy, and DEAF-1, which are the three most characterized proteins that contain the MYND domain. Only a small number of MYND domain containing proteins have been identified and they are involved in various biological processes such as cell proliferation, apoptosis, adhesion, migration, and tumorigenesis and oxygen homeostasis. MYND domain typically binds a proline-rich motif in their interacting partners, however they have different binding specificities.

Table S9. Eukaryotic linear motifs and their functions (continued).

ELM	Evidence Class	Logical	Reability	Code	Probability	ELM Description	Functional Description
LIG_SUMO_SBM_2	Experimental	TP	Likely	m	0.018383	Minimal region common to all known SBM instances and required for SBM function is the hydrophobic patch consisting of 3 hydrophobic residues (I, V, or L) or 4 residues consisting of 3 hydrophobic residues (I,V, or L) and a variable residue at 2nd or 3rd positions. Contrary to LIG_SUMO_SBM_1, in the inverted SBM, the hydrophobic core is preceded by a variable length stretch of phosphorylatable residues (mainly Serine) and acidic residues. Both the phoshorylatable residues (when phosphorylated) and the acidic residues increase the affinity of binding to SUMO due to interactions with basic residues of SUMO on SBM interaction interface. Inverted SBM forms a beta sheet pairing with the second beta sheet of SUMO in anti-parallel orientation (eg RanBP2 (P49792)) (Reverter,2005).	Non-covalent binding to SUMO proteins is mediated via SUMO-binding motif (SBM). Sumo-interacting proteins predominantly function in the nucleus. SBM is essential for a variety of cellular processes including transcriptional regulation, sub-nuclear localization, nuclear body assembly, and anti-viral response. Viral proteins are also known to utilize such processes via their SBMs upon host cell invasion.
LIG_WD40_WDR5_1	Experimental	TP	Certain	n	0.0001462	The conserved WDR5-binding motif in the unstructured acidic C-terminal tail region of RbBP5 interacts on the edge of the beta-propeller between blades 5-6 on the opposite face of the WDR5 protein where MLL1 binds with the Win motif (LIG_WD40_WDR5_WIN_1, LIG_WD40_WDR5_WIN_2, LIG_WD40_WDR5_WIN_3). The motif is negatively charged due to multiple acidic residues, while the RbBP5-binding site is positively charged, allowing accommodation of the peptide. The acidic residues include one aspartate between the two conserved hydrophobic positions, and up to four consecutive aspartate and/or glutamate residues (generally a minimum of two acidic residues randomly distributed at these four positions is observed) N-terminal to the first conserved hydrophobic position. After binding there is a negatively charged complex. The RbBP5-binding site has hydrophobic features allowing two conserved valine residues to bind into two pockets. The first pocket binds Val-375 in human RbBP5, and is made up of Tyr-228 and Leu-240 and the aliphatic part of the Lys-250 side chain. The second pocket is formed by Phe-266, Val-268 and Leu-288 and interacts with Val-377. Polar interactions between the WDR5 residues Asn-225 and Gln-289 and the main chain help stabilize the binding of the valine residues into the hydrophobic pockets. Not all acidic residues contributing to the negativity of the peptide interact with the WDR5 surface as they are orientated away from the protein, however they may be of importance due to their negativity (Odho,2010) (2XL2). The motif is highly conserved among eukaryotes, especially amongst vertebrates and mammals, however among fungi some variances can be observed, which are captured in a separate motif specific for fungal species (LIG_WD40_WDR5_2).	In the nuclei of eukaryotic cells, DNA is complexed with histones into nucleosomes. Post-translational modification of histones regulates their interactions with DNA and other nuclear proteins, and is important for the control of cellular processes such as gene transcription, cell cycle progression and DNA repair. One important modification is the methylation of H3 histones at lysine 4, which is located in the intrinsically disordered N-terminal H3 region. Methylation of H3K4 is catalyzed by members of the Set1/MLL protein family, whose activity depends on their assembly in a multi-protein histone methylation complex. The WD40 repeat domain protein WDR5 plays a key role in H3K4 methylation by acting as a scaffold protein for the assembly of the core histone methylation complex, which is conserved through evolution. The recruitment of different complex subunits by WDR5 depends on distinct motifs in WDR5-binding partners, including the catalytic Set1/MLL subunits and the accessory protein RbBP5.
MOD_CK1_1	Experimental	TP	Certain	o	0.0170407	CK1 Phosphorylation site	Motif recognised by CK1 for Ser/Thr phosphorylation
MOD_CK2_1	Experimental	TP	Certain	p	0.0145681	The main determinant of CK2 phosphorylation specificity is a negative charge 3 positions after the modification residue.	Motif recognised by CK2 for Ser/Thr phosphorylation
MOD_GSK3_1	Experimental	TP	Certain	q	0.0267866	The GSK3 phosphorylation recognition site is based primarily on vertebrate data. GSK3-family kinases are widespread in Eukaryota but in most cases the specificity has not been confirmed.	Site recognised by GSK3 for Ser/Thr phosphorylation.
MOD_NEK2_1	Experimental	TP	Certain	r	0.0097983	The optimal substrate motif of NEK2 was determined by positional scanning oriented peptide library screening (PS-OPLS) (Alexander,2011). The substrate motif targeted by NEK2 for phosphorylation shows the strongest amino acid selectivity in the -3 and +2 positions (relative to the Ser/Thr residue that is phosphorylated by NEK2). Having a preferred residue in the -3 position might compensate for the occurrence of less favorable residues in the +1 and +2 positions and vice versa. To encode this information, two variants of the motif have been defined, based on the selectivity for particular residues observed in the PS-OPLS experiment (Alexander,2011). The first variant of the NEK2 substrate phosphorylation motif contains Phe, Leu or Met in the -3 position. The occurrence of one of these residues, which are highly preferred to other hydrophobic residues, might compensate for less favored amino acids in the +1 and +2 positions. Hydrophilic amino acids as well as Ile and Val are strongly disfavored in the -3 position. In the -2 position, all amino acids except Pro are tolerated, although there is a slight preference for basic and hydrophobic residues. Similarly, in the -1 position there is no strong selectivity except for a strong discrimination against Pro. Also in the +1 position Pro is disfavored, together with the acidic Glu and Asp residues. The strong selection against Pro in this position allows discrimination against Cdk1 mitotic kinase phosphorylation sites. In the +2 position, the acidic residues Glu and Asp are not allowed, while a Pro residue is again accepted in this position. Although there seems to be a preference for particular hydrophobic residues in the +1 position and for specific amino acids in the +2 position, the occurrence of less favored residues in these two positions is allowed in this variant of the motif, as this might be compensated by the presence of the strongly selected Phe, Leu or Met in the -3 position.	The NEK protein kinases are Never in mitosis A (NimA)-related kinases that belong to the NEK Serine/Threonine protein kinase family. NEKs have been identified in many Eukaryotes, where they play a critical role in cell cycle control. The closest mammalian NimA homologue NEK2 is a core component of the human centrosome and its activity and expression peak in S and G2 phase, during which it interacts with and phosphorylates several centrosomal proteins. NEK2 has many cell cycle-related functions, including cell cycle progression, spindle pole formation, microtubule anchoring, centriolar cohesion, cilia formation and chromatin condensation.
MOD_PIKK_1	Experimental	TP	Certain	s	0.0092301	The PIKK family member proteins specifically phosphorylate the (ST)Q motif in their substrates. The glutamine adjacent to the target serine-threonine is critical for the substrate recognition.	The phosphoinositide-3-OH-kinase related kinases (PIKKs) are atypical protein kinases exclusive to eukaryotes. The PIKK members are large proteins with Ser/Thr kinase activity serving important roles in DNA repair and DNA damage checkpoints. The three PIKK proteins with repair and checkpoint functions in mammalian cells are: DNA-PK (DNA-dependent protein kinase), ATM (ataxia telangiectasia mutated), and ATR (ATM and Rad3 related).
MOD_PKA_1	Experimental	TP	Certain	t	0.0023151	The optimal phosphorylation site preference for PKA is RR.(ST) - Lysine in the basic sites may weaken the prefence. This site is also targeted by other basophilic kinases of the AGC group, including PKG and PKC isoforms. AGC group kinases do not tolerate Pro at position +1. Lys at the Arg positions may be allowed in some weaker sites. Some specificity determinants may be present at the less conserved non-basic sites.	Motifs phosphorylated by a subset of AGC group kinases including PKA that all have similar sequence specificity.
MOD_PKA_2	Experimental	TP	Certain	u	0.0094575	Secondary preference for PKA-type AGC kinase phosphorylation with a single Arg at p-2. This motif is probably more often targeted by other basophilic kinases of the AGC group, including PAK1 and PKC isoforms: These kinases actually show a stronger preference at p-2 than PKA, which has the strongest basophilic preference at p-3. AGC group kinases do not tolerate Pro at position +1. It is likely that some specificity determinants distinguishing among these kinases may be present at the less conserved non-basic sites.	Motifs phosphorylated by a subset of AGC group kinases including PKA that all have similar sequence specificity.
MOD_PLK	Experimental	TP	Certain	v	0.0060146	Site phosphorylated by the Polo-like kinase.	Site recognised and phosphorylated by the Polo-like kinase.

Table S9. Eukaryotic linear motifs and their functions (continued).

ELM	Evidence Class	Logical	Reability	Code	Probability	ELM Description	Functional Description
MOD_ProDKin_1	Experimental	TP	Certain	w	0.0154332	(S/T)P site at which Pro-directed kinases such as P38 MAP Kinase phosphorylate a Ser/Thr residue in various signal transduction pathways. These kinases require a docking motif to be activated, so the site is not meaningful by itself. MAP Kinase homologues are found widely in eukaryotes, including protozoa, but experimental data is from higher eukaryotes, mainly metazoa.	Site at which MAP Kinase phosphorylates substrates.
DOC_ANK_TNKS_1	Experimental	TP	Certain	x	0.0003538	Tankyrase1 and Tankyrase 2 are closely related poly (ADP-ribose) polymerases. They have both enzymatic and scaffolding activities. Tankyrases are multidomain proteins. The C-terminal PARP domain catalyzes the poly-(ADP-ribosyl)ation of substrate proteins, whereas the ankyrin repeat domain interacts with the substrates and brings them to PARP domain. There are five ankyrin triple repeat clusters (ARCs) present in Tankyrase and, except ARC3, all are predicted to act as independent binding unit. The ARCs interact with substrates through their tankyrase binding motifs. All ARCs having similar substrate recognition abilities and the overall binding mode of substrates to an ARC of Tankyrase is highly conserved, with the binding peptide lying in a surface groove. Each ARC is able to bind the same set of proteins with different binding affinity. So they are redundant in function. The Tankyrase binding motif is usually depicted as RxxPDG. The arginine and glycine residues at positions 1 and 6 are highly conserved acting as critical anchor residues of the motif. The R enters the deepest part of the groove making charged interactions. The G is sandwiched in the narrowest part of the groove. The P and D positions are more variable. The motif is experimentally verified in nearly 17 proteins including 3BP2, AXIN1, TRF1, IRAP and NUMA1.	Tankyrases belong to the poly (ADP-ribose) polymerase (PARP) family of proteins, which function by catalyzing the covalent linkage of ADP-ribose polymers onto target proteins and thereby regulating their ubiquitylation, stability and function. The human genome encodes two similar tankyrases. TNKS and TNKS2 both recruit a variety of substrates with a broad range of functions. Each shows some variation in their conservation and tissue expression. Moreover both Tankyrases contain N-terminal ankyrin repeats. These ankyrin repeats form five domains known as ankyrin repeat clusters (ARCs) and each can serve as a discrete binding site for its binding partners. Tankyrase plays important roles in many biological functions including telomere length regulation, insulin signalling and centrosome function. Dysregulation of 3BP2 recognition by tankyrase causes a human disease knows as cherubism. Tankyrase is also considered to be a potential candidate for a telomere-directed anticancer target.
MOD_PKB_1	Experimental	TP	Certain	y	0.0006034	Phosphorylation site preference R.R.(ST) targeted by some basophilic kinases of the AGC group, including PKB and p70S6K. AGC group kinases do not tolerate Pro at position +1. Lys at the Arg positions may be allowed in some weaker sites. Some specificity determinants may occur at the less conserved non-basic sites: e.g Phe at position +2 is reported to be an optimal PKB site but a poor one for p60S6K.	Motif phosphorylated by a subset of AGC group kinases including PKB that all have similar sequence specificity.
LIG_MAD2	Experimental	TP	Certain	z	0.0001011	Mitotic spindle checkpoint protein MAD2 binding motif	Mitotic spindle checkpoint protein MAD2 binding motif

CLV: Cleavage Sites; DEG: Degradation sites; DOCK: Docking sites; Ligand binding sites; MOD: post-translational modification sites;TP: True Positive.
