# Supplementary material for: FOXP in Tetrapoda: Intrinsically Disordered Regions, Short Linear Motifs and their evolutionary significance
Source: Genet Mol Biol. 2017 Mar 2;40(1):181–90. doi: 10.1590/1678-4685-GMB-2016-0115 (PMC5409772; doi:10.1590/1678-4685-GMB-2016-0115)
Supplement: Supplementary file 8 [file 1415-4757-gmb-1678-4685-GMB-2016-0115-Suppl14.pdf]

Table S7. Estimated parameters under different codon substitution models for forkhead P sub-family genes[1].

	Model	*dN/dS*	Estimated parameters	Sites with ω>1[2]	***ℓ***	*P value*
FOXP1	M1a: neutral	0.0731	p_0 = 0.95618, (p_1 = 0.04382) (ω_0 = 0.03066), (ω_1 = 1.00000)		−13857.331728	1
	M2a: selection	0.0731	p_0 = 0.95618, p_1 = 0.04382, (p_2 = 0.00000) (ω_0 = 0.03066), (ω_1 = 1.00000), ω_2 = 11.32611		−13857.331728	
	M8a: β & ω=1	0.0553	p_0 = 0.99268, (p1 = 0.00732), p = 0.15813 q = 2.81242, ω = 1.00000		−13799.608758	1
	M8: β & ω	0.0553	p_0 = 0.99268, (p1 = 0.00732), p = 0.15813, q = 2.81243, ω = 1.00000		−13799.608758	
FOXP2	M1a: neutral	0.0615	p_0 = 0.95735, (p_1 = 0.04265) (ω_0 = 0.01965), (ω_1 = 1.00000)		−12558.167089	0.9295
	M2a: selection	0.0628	p_0 = 0.95601, p_1 = 0.01529, (p_2 = 0.02870) (ω_0 = 0.01965), (ω_1 = 1.00000), ω_2 = 1.00000		−12558.093967	
	M8a: β & ω=1	0.0458	p_0 = 0.99999, (p_1 = 0.00001), p = 0.08897 q = 1.61951, ω = 1.00000		−12524.222980	1
	M8: β & ω	0.0455	p_0 = 0.99999, (p1 = 0.00001), p = 0.09061, q = 1.66060, ω = 1.00001		−12524.233000	

[1]p_0 = proportion of sites where ω < 1, p_1 = proportion of sites where ω = 1, and p_2 = proportion of sites where ω > 1 (selection models only), ω_0 < 1 (negative selection), ω_1 = 1 (neutral selection), and ω_2 > 1 (positive selection), likelihood ratio tests were performed between neutral models (M1a - Nearly Neutral, and M7 - Beta) and models that identify positive selection (M2a - Selection, and M8 – Beta + Selection), the comparisons between M1 vs. M2 and M7 vs. M8 have 2 degrees of freedom. Parentheses indicate fixed parameters.

[2]Position and protein IUPAC code.

Table S7. Estimated parameters under different codon substitution models for forkhead P sub-family genes[1] (continued).

	Model	*dN/dS*	Estimated parameters	Sites with ω>1[2]	ℓ	*P value*
FOXP3	M1a: neutral	0.1834	$p_0 = 0.89272$, ($p_1 = 0.10728$) ($\omega_0 = 0.08525$), ($\omega_1 = 1.00000$)		−12510.124897	1
	M2a: selection	0.1834	$p_0 = 0.89272$, $p_1 = 0.04720$, ($p_2 = 0.06008$) ($\omega_0 = 0.08525$), ($\omega_1 = 1.00000$), $\omega_2 = 1.00000$		−12510.124897	
	M8a: β & ω=1	0.1518	$p_0 = 0.95683$, ($p_1 = 0.04317$), $p = 0.48347$ $q = 3.64447$, $\omega = 1.00000$		−12424.107542	0.9137
	M8: β & ω	0.1532	$p_0 = 0.96152$, ($p_1 = 0.03848$), $p = 0.47274$, $q = 3.45516$, $\omega = 1.06357$	183S, 184A, 194V, and 301V	−12424.017242	
FOXP4	M1a: neutral	0.0487	$p_0 = 0.97844$, ($p_1 = 0.02156$) ($\omega_0 = 0.02778$), ($\omega_1 = 1.00000$)		−18552.897747	1
	M2a: selection	0.0487	$p_0 = 0.97843$, $p_1 = 0.02157$, ($p_2 = 0.00000$) ($\omega_0 = 0.02778$), ($\omega_1 = 1.00000$), $\omega_2 = 6.24385$		−18552.897760	
	M8a: β & ω=1	0.0384	$P_0 = 0.99789$, ($p_1 = 0.00211$), $p = 0.23183$ $q = 5.59727$, $\omega = 1.00000$		-18335.456738	1
	M8: β & ω	0.0392	$p_0 = 0.99999$, ($p_1 = 0.00001$), $p = 0.22328$ $q = 4.99479$, $\omega = 2.82598$		−18340.025059	

[1]p_0 = proportion of sites where $\omega < 1$, p_1 = proportion of sites where $\omega = 1$, and p_2 = proportion of sites where $\omega > 1$ (selection models only), $\omega_0 < 1$ (negative selection), $\omega_1 = 1$ (neutral selection), and $\omega_2 > 1$ (positive selection), likelihood ratio tests were performed between neutral models (M1a - Nearly Neutral, and M7 - Beta) and models that identify positive selection (M2a - Selection, and M8 – Beta + Selection), the comparisons between M1 vs. M2 and M7 vs. M8 have 2 degrees of freedom. Parentheses indicate fixed parameters.
[2]Position and protein IUPAC code.
