# Supplementary material for: FOXP in Tetrapoda: Intrinsically Disordered Regions, Short Linear Motifs and their evolutionary significance
Source: Genet Mol Biol. 2017 Mar 2;40(1):181–90. doi: 10.1590/1678-4685-GMB-2016-0115 (PMC5409772; doi:10.1590/1678-4685-GMB-2016-0115)
Supplement: Supplementary file 9 [file 1415-4757-gmb-1678-4685-GMB-2016-0115-Suppl15.pdf]

Table S8. Branch site model for *FOXP* genes.

		Model Clade D (free-ratio model, admit selection)				M1a (neutral)		Probability
		Proportion	Clade 1[b]	Clade 2[f]	LogL	Proportion	LogL	M1a vs. Clade
FOXP1[2]	Mammals[f] vs. non-mammals[b]	P_0 = 0.70045	ω_0 = 0.00176	ω_0 = 0.00176		P_0 = 0.93230		
		P_1 = 0.24561	ω_1 = 0.13116	ω_1 = 0.13116	−15531.277393	P_1 = 0.06770	−15594.616874	1
		P_2 = 0.05394	ω_2 = 0.68705	ω_2 = 0.60693		ω_0 = 0.03010		
						ω_1 = 1.00000		
FOXP4	Mammals[f] vs. non-mammals[b]	P_0 = 0.70046	ω_0 = 0.00627	ω_0 = 0.00627		P_0 = 0.95450		
		P_1 = 0.25887	ω_1 = 0.11160	ω_1 = 0.11160	−22189.482510	P_1 = 0.04550	−22463.434676	<0.001
		P_2 = 0.04067	ω_2 = 0.18012	ω_2 = 0.66102		ω_0 = 0.03293		
						ω_1 = 1.00000		

[1]P = Proportion of codons in each ω class, where ω = dN/dS (non-synonymous/synonymous rate ratio), two degrees of freedom were considered.
[2]For such analysis in FOXP1, *Xenopus sp.* was excluded. Mammals and primates are respective to Clade 2.
[f] = foreground clade, [b] = background
