# Supplementary material for: FOXP in Tetrapoda: Intrinsically Disordered Regions, Short Linear Motifs and their evolutionary significance
Source: Genet Mol Biol. 2017 Mar 2;40(1):181–90. doi: 10.1590/1678-4685-GMB-2016-0115 (PMC5409772; doi:10.1590/1678-4685-GMB-2016-0115)
Supplement: Supplementary file 10 [file 1415-4757-gmb-1678-4685-GMB-2016-0115-Suppl12.pdf]

Table S6.3. Whole protein comparison for FOXP3 linear motifs content.

**Linear Motfis**	CLV_C14_Caspase3-7	DEG_SCF_FBW7_1	DOC_CKS1_1	DOC_CYCLIN_1	DOC_MAPK_1	DOC_PP2B_1	DOC_PP2B_2	DOC_USP7_1	DOC_WW_Pin1_4	LIG_14-3-3_2	LIG_14-3-3_3	LIG_BRCT_BRCA1_1	LIG_FHA_1	LIG_FHA_2	LIG_MYND_1	LIG_NRBOX	LIG_PTAP_UEV_1	LIG_RRM_PRI_1	LIG_SUMO_SBM_1	LIG_SUMO_SBM_2	MOD_CDK_1	MOD_CK1_1	MOD_CK2_1	MOD_GSK3_1	MOD_NEK2_1	MOD_NEK2_2	MOD_PIKK_1	MOD_PKA_1	MOD_PKA_2	MOD_PLK	MOD_ProDKin_1	TRG_NES_CRM1_1	TRG_NLS_MonoCore_2	TRG_NLS_MonoExtN_4	Total
Homo sapiens	2	2				1	3	7	6	1	1		2	2	1	1	2			1		6	2	9	3	1	3	2	2	1	6		1	1	69
Homo neanderthalensis	2	2			1		2	7	5	1	1		1	2	1		2					4	2	9	3	1	3	2	2	1	5	1	1	1	62
Pan troglodytes	2					1	3	6	5	1	1		2	2	1	1	1			1		6	2	8	3	1	3	2	2	1	5		1	1	62
Pan paniscus	2					1	3	6	5	1	1		2	2	1	1	1			1		6	2	8	3	1	3	2	2	1	5		1	1	62
Gorilla gorilla	2				1	1	3	6	5	1	1		2	2	1	1				1		6	2	9	3	1	3	2	2	1	5		1	1	63
Pongo abelii	2					1	3	6	5	1	1		3	2	1	1	1			1		7	2	8	3	1	3	2	2	1	5		1	1	64
Pongo pygmaeus	2					1	3	6	5	1	1		3	2	1	1	1			1		7	2	8	3	1	3	2	2	1	5		1	1	64
Nomascus leucogenys	2					1	3	5	5	1	1		3	2	1	1	1			1		6	2	9	3	1	3	2	2	1	5		1	1	63
Hylobates lar	2					1	3	6	5	1	1		3	2	1	1	2			1		6	2	9	3	1	3	2	2	1	5		1	1	65
Macaca mulatta	2			1		1	3	6	6	1	1		2	2	1	1	1		1	2		7	2	8	4	1	3	2	2	1	6	1	1	1	70
Papio anubis	2					1	3	6	5	1	1		2	2	1	1	1			1		7	2	8	3	1	3	2	2	1	5		1	1	63
Chlorocebus sabaeus	2					1	3	6	5	1	1		2	2	1	1	1		1	1		7	2	8	3	1	3	2	2	1	5	1	1	1	65
Saimiri boliviensis	2	1				1	3	6	6	1	1		3	2	1	1	1			1		8	2	11	1	1	3	2	2	2	6		1	1	70
Callithrix jacchus	2	1				1	2	6	6	1	1		3	2	1	1	1			1		7	2	10	2	1	3	2	2	2	6		1	1	68
Tarsius syrichta	2	1				1	2	4	7	1			3	1	1	1	1			3		5	1	10	3	1	3	2	1	1	7		1	1	64
Galeopterus variegatus	2			1		1	3	5	7	1			3	2	1	1			1	1		5	2	7	3	1	3	2	2	1	7		1	1	64
Tupaia chinensis	2		1		1	1	3	5	7	1	1		4	2		1	1			4		14	2	16	4	1	3	2	3	1	7		1	1	89
Mus musculus	2					1	4	3	4		1		2	1	1	1				1	1	3	2	6	4	1	3	2	1		4		1	1	50
Rattus norvegicus	2					1	4	5	5		2		2	2	2	1				1	1	3	3	8	5	2	3	2	1		5		1	1	62

Table S6.3. Whole protein comparison for FOXP3 linear motifs content (continued).

**Linear Motfis**	CLV_C14_Caspase3-7	DEG_SCF_FBW7_1	DOC_CKS1_1	DOC_CYCLIN_1	DOC_MAPK_1	DOC_PP2B_1	DOC_PP2B_2	DOC_USP7_1	DOC_WW_Pin1_4	LIG_14-3-3_2	LIG_14-3-3_3	LIG_BRCT_BRCA1_1	LIG_FHA_1	LIG_FHA_2	LIG_MYND_1	LIG_NRBOX	LIG_PTAP_UEV_1	LIG_RRM_PRI_1	LIG_SUMO_SBM_1	LIG_SUMO_SBM_2	MOD_CDK_1	MOD_CK1_1	MOD_CK2_1	MOD_GSK3_1	MOD_NEK2_1	MOD_NEK2_2	MOD_PIKK_1	MOD_PKA_1	MOD_PKA_2	MOD_PLK	MOD_ProDKin_1	TRG_NES_CRM1_1	TRG_NLS_MonoCore_2	TRG_NLS_MonoExtN_4	Total
Cricetulus griseus	2					1	4	5	5		1		2	2	1	1				1	1	2	3	5	5	2	3	1	1		5		1	1	55
Octodon degus	2		2	1		1	4	8	9	2	2	1	3	2	1	1	1		2	5		5	3	9	4	1		1	2	1	9		1	1	84
Oryctolagus cuniculus	2					1	3	5	7	1	1		4	2	1	1	1			3		6	3	9	3	2	3	2	4	2	7		1	1	75
Ochotona princeps	2	1				1	3	9	8	1	1		4	2	1	1	1			3	1	10	3	15	2	2	3	2	4	2	8		1	1	92
Physeter catodon	1			1		1	3	6	9	1	2		2	2	1	1			2	2		5	2	10	2	1	2	2	2	1	9		1	1	72
Orcinus orca	1			1		1	3	6	8	1	2		2	2	1	1			2	2		5	2	9	2	1	2	2	2	1	8		1	1	69
Bos taurus	2			1		1	3	5	10	1	1		2	2	1	1			2	2		6	2	13	3	1	2	2	2	1	10		1	1	78
Camelus ferus	2			1		1	3	5	7	1	1		2	3	1	1		1	1	2		5	3	8	2	1	3	2	2	1	7		1	1	68
Vicugna pacos	2			1		1	3	5	7	1	1		2	3	1	1		1	1	2		5	3	8	2	1	3	2	2	1	7		1	1	68
Ceratotherium simum simum	3	3					3	8	9	1	1		2	2	1	1	1		1	1		6	2	15	3	1	2	2	2	2	9		1	1	83
Equus caballus	1					1	3	7	9	1			2	2	1	1	2		1	1		6	3	10	4	1	2	2	2	1	9		1	1	74
Leptonychotes weddellii	1		1			1	3	7	10		1		2	3	1	1	1		1	1	1	4	4	9	2	1	2	2	2		10		1	1	73
Odobenus rosmarus divergens	1		2			1	3	7	11	1	1		2	2	1	1	1	1	1	2	1	5	3	12	3	1	2	2	1	1	11		1	1	82
Mustela putorius furo	1		1			1	3	8	11		1		3	2	1	1	1	1	1	2	1	8	3	16	3	1	2	2	1		11		1	1	88
Ailuropoda melanoleuca	1		1			1	3	9	10	1	1		2	2	1	1			1	1	1	9	3	15	4	1	2	2	1	1	10		1	1	86
Canis lupus familiaris	1		1			1	3	8	9	1			2	2	2	1	1		1	1	1	6	3	13	3	1	2	2	2	1	9		1	1	79
Panthera tigris	1		2			1	3	7	10	1			2	2	1	1	1		1	1	1	6	3	10	3	1	2	2	2	1	10		1	1	77
Felis catus	1		1			1	3	6	10	1			2	1	1	1	1		1	1	1	6	2	12	3	1	2	2	2	1	10		1	1	75
Myotis brandtii	1			1			4	5	6		1		2	2	1	1			2	2		4	2	10	3	1	2	2	2	1	6		1	1	63

Table S6.3. Whole protein comparison for FOXP3 linear motifs content (continued).

**Linear Motfis**	CLV_C14_Caspase3-7	DEG_SCF_FBW7_1	DOC_CKS1_1	DOC_CYCLIN_1	DOC_MAPK_1	DOC_PP2B_1	DOC_PP2B_2	DOC_USP7_1	DOC_WW_Pin1_4	LIG_14-3-3_2	LIG_14-3-3_3	LIG_BRCT_BRCA1_1	LIG_FHA_1	LIG_FHA_2	LIG_MYND_1	LIG_NRBOX	LIG_PTAP_UEV_1	LIG_RRM_PRI_1	LIG_SUMO_SBM_1	LIG_SUMO_SBM_2	MOD_CDK_1	MOD_CK1_1	MOD_CK2_1	MOD_GSK3_1	MOD_NEK2_1	MOD_NEK2_2	MOD_PIKK_1	MOD_PKA_1	MOD_PKA_2	MOD_PLK	MOD_ProDKin_1	TRG_NES_CRM1_1	TRG_NLS_MonoCore_2	TRG_NLS_MonoExtN_4	Total
Eptesicus fuscus	1			1			3	5	6		2	2	4	3	1	1			2	2		2	3	12	3	1	2	2	1	2	6		1	1	69
Pteropus alecto	2					1	3	5	7	1	1		2	2		1	1		2	3		5	2	10	4	1	3	2	2	1	7		1	1	70
Erinaceus europaeus	1		1	1		1	2	8	7				4	2	1	1				1	1	7	2	12	2	1	3	1	1	1	7		1	1	70
Sorex araneus						1	2	5	9	1	3	2	2	2	1	1			1	1	1	8	3	8	3		3	2	3	1	9		1	1	74
Condylura cristata	1					1	2	4	8	1	1		2	2	1	1	1		1	1	1	5	2	8	3	1	3	2	2	1	8		1	1	65
Dasypus novemcinctus	2				1		3	6	7	1			2	2	1	1			1	1		7	2	6	3	1	3	2	3	1	7		1	1	65
Echinops telfairi		1	1			1	4	6	8		2	1	3	1	2	1			1	1		10	2	13	4	1	2	2	2		8		1	1	79
Chrysochloris asiatica	1		1			1	4	4	8	1	1		2	2	2	1			1	1	1	6	3	11	3	1	2	2	2	1	8		1	1	72
Elephantulus edwardii	1		1	1			2	7	8	1	1		3	2	1	1			2		1	4	2	10	3	1	2	2	2	1	8	1	1	1	70
Orycteropus afer afer	1	1				1	3	7	8	1	1		2	2	2	1			1	1		6	2	14	3	1	2	2	1	1	8	1	1	1	75
Trichechus manatus latirostris	1			1		1	3	6	8		1		2	2	2				1	1		6	2	9	3	1	3	1	2		8	1	1	1	67
Loxodonta africana	1			1			3	3	5	1	1	1	2	2	1					1		7	2	9	4	1	3	2	2	1	5	1	1	1	61
Monodelphis domestica	1	2	1	4			3	8	6		1	1	8	2		1			1			3	2	14	3		1	1	3	1	6		2	2	77
Ornitorhynchus anatinus	2	2	1	2	1		4	6	12		4	3	4	2		1			1	1		12	5	20	5	1	5	2	3		12			1	112
