# Supplementary material for: FOXP in Tetrapoda: Intrinsically Disordered Regions, Short Linear Motifs and their evolutionary significance
Source: Genet Mol Biol. 2017 Mar 2;40(1):181–90. doi: 10.1590/1678-4685-GMB-2016-0115 (PMC5409772; doi:10.1590/1678-4685-GMB-2016-0115)
Supplement: Table S2.2 [file 1415-4757-gmb-1678-4685-GMB-2016-0115-Suppl03.pdf]

Table S2.2. Ordered regions for FOXP1 orthologues.

Species	Structured Region				
Homo sapiens			220-234	302-364	440-574
Pan troglodytes			220-234	302-364	440-574
Gorilla gorilla			220-234	302-364	440-574
Pongo abelii			220-234	302-364	440-574
Nomascus leucogenys			220-234	302-364	440-574
Macaca mulatta			220-234	302-364	440-574
Papio anubis			220-234	302-364	440-574
Chloroscebus sabaeus			220-234	302-364	440-574
Saimiri boliviensis boliviensis		52-55	219-233	302-363	439-573
Callithrix jacchus			220-234	302-359	440-574
Galeopterus variegatus		52-57	220-234	302-364	440-587
Tupaia chinensis			220-235	301-364	440-573
Mus musculus			218-230	300-362	438-572
Cricetulus griseus			226-240	308-370	446-580
Rattus novergicus			254-268	336-398	474-610
Oryctolagus cuniculus			228-241	310-372	448-582
Ochotona princeps			225-239	307-369	445-578
Octodon degus			227-238	309-369	445-579
Eptesicus fuscus			220-228	299-362	438-578
Myotis brandtii			222-230	301-364	440-574
Pteropus alecto		49-62	218-232	300-362	438-572
Ceratotherium simum simum	16-28	37-73	222-236	305-366	442-576
Felis catus	16-28	39-72	222-236	304-366	442-576
Panthera tigris	16-28	39-72	222-236	304-366	442-576
Odobemus rosmarus divergens		53-57	220-233	301-364	440-574
Erinaceus europaeus			217-231	298-361	437-571
Physeter catodon		52-56	216-229	298-354	436-572
Camelus ferus	13-29	36-69	222-360		442-575
Vicugna pacos		52-56	220-233	302-358	440-573
Echinops telfairi			220-236	301-364	441-574
Trichechus manatus			229-244	310-367	449-581
Orycteropus afe afer			220-235	301-364	441-574
Elephantulus edwardii			226-239	307-370	447-577
Chrysochloris asiatica			224-240	306-369	446-579
Condylura cristata			224-238	306-368	444-584
Loxodonta africana			227-241	308-365	447-581
Monodelphis domestica		52-63	218-228	302-364	441-576
Ficedula albicollis		47-57	225-237	308-364	445-581
Taeniopygia guttata		42-79	253-265	336-392	473-609
Falco peregrinus		44-59	227-239	310-366	447-583
Calypte anna			216-228	299-355	436-572
Aptenodytes forsteri		47-57	227-239	310-366	447-588
Zonotrichia albicollis		47-57	227-238	309-365	447-583
Manacus vitellinus		51-60	227-239	310-366	447-583
Serinus canaria		52-58	227-239	310-366	447-583
Melopsittacus undulatus		47-56	226-238	308-364	446-582

Table S2.2. Ordered regions for FOXP1 orthologues (continued).

Species	Structured Region			
Gallus gallus	47-56	229-241	312-368	449-592
Anas platyrhynchos		226-238	309-365	446-582
Python bivittatus	47-56	230-244	311-378	450-582
Anolis carolinensis		230-246	312-364	450-582
Chelonia mydas	47-58	217-232	302-364	437-580
Pelodiscus sinensis	44-62	217-230	300-356	435-580
Xenopus laevis		121-161	202-258	341-473
