# Supplementary material for: FOXP in Tetrapoda: Intrinsically Disordered Regions, Short Linear Motifs and their evolutionary significance
Source: Genet Mol Biol. 2017 Mar 2;40(1):181–90. doi: 10.1590/1678-4685-GMB-2016-0115 (PMC5409772; doi:10.1590/1678-4685-GMB-2016-0115)
Supplement: Table S3.2 [file 1415-4757-gmb-1678-4685-GMB-2016-0115-Suppl05.pdf]

Table S3.2. Ordered Regions For FOXP2 orthologues.

Species	Structured Region			
Homo sapiens		259-280	341-390	484-628
Pan troglodytes		259-280	342-391	485-629
Gorilla gorilla		257-278	339-388	482-626
Pongo abelii		257-288	339-388	468-626
Nomascus leucogenys		258-278	339-388	482-626
Macaca mulatta		258-289	340-389	483-627
Papio anubis		258-279	340-389	483-627
Chloroscebus sabaeus		258-279	340-389	483-618
Saimiri boliviensis boliviensis		258-279	340-389	483-627
Callithrix jacchus		257-278	339-388	482-617
Galeopterus variegatus	87-99	275-296	357-406	500-644
Tupaia chinensis		258-279	340-389	483-627
Mus musculus		258-289	340-389	483-627
Cricetulus griseus		262-282	343-392	486-630
Rattus novergicus		253-273	336-385	479-627
Oryctolagus cuniculus		260-281	342-391	485-629
Ochotona princeps		251-271	332-381	475-610
Octodon degus		263-280	344-393	487-631
Eptesicus fuscus		258-281	341-389	483-618
Myotis brandtii		257-280	340-389	483-618
Pteropus alecto		261-282	344-392	486-630
Ceratotherium simum simum		257-278	339-388	482-627
Felis catus		251-272	333-382	476-620
Panthera tigris		231-252	315-364	458-602
Odobemus rosmarus divergens		253-274	335-384	478-622
Erinaceus europaeus		252-271	335-383	477-621
Physeter catodon		252-274	334-383	477-621
Camelus ferus		254-275	336-385	479-614
Vicugna pacos		254-275	336-385	479-614
Echinops telfairi		260-277	341-390	484-615
Trichechus manatus		268-289	350-399	493-637
Orycteropus afe afer		256-277	339-387	481-637
Elephantulus edwardii		256-277	338-387	480-625
Chrysochloris asiatica		258-278	339-388	482-626
Condylura cristata		258-279	340-389	483-627
Loxodonta africana		255-276	335-386	480-624
Monodelphis domestica		253-272	335-384	478-622
Ficedula albicolis		274-293	358-405	499-643
Taeniopygia guttata		255-274	337-386	480-624
Falco peregrinus		270-288	351-395	494-638
Calypte anna		272-291	354-404	496-640

Table S3.2. Ordered Regions For FOXP2 orthologues (continued).

Species	Structured Region				
Aptenodytes forsteri	252-271	334-383			477-621
Zonotrichia albicollis	253-272	335-384			478-622
Manacus vitellinus	250-273	334-381			476-620
Serinus canaria	256-275	338-387			481-625
Melopsittacus undulatus	253-272	335-384			478-622
Gallus gallus	252-271	334-383			477-621
Anas platyrhynchos	256-275	338-387			481-625
Python bivittatus	248-267	330-379			473-624
Anolis carolinensis	265-284	347-391			491-634
Chelonia mydas	250-269	332-380			475-619
Pelodiscus sinensis	249-268	331-380			474-619
Xenopus laevis	250-273	333-381	461-466	475-631	655-668
