# Supplementary material for: FOXP in Tetrapoda: Intrinsically Disordered Regions, Short Linear Motifs and their evolutionary significance
Source: Genet Mol Biol. 2017 Mar 2;40(1):181–90. doi: 10.1590/1678-4685-GMB-2016-0115 (PMC5409772; doi:10.1590/1678-4685-GMB-2016-0115)
Supplement: Table S4.1 [file 1415-4757-gmb-1678-4685-GMB-2016-0115-Suppl06.pdf]

Table S4.1. Disorder Proportion for FOXP3 orthologues.

Species	Disorder Proportion	AA	Order	Class
Homo sapiens	0.280742459	431	Primates	Mammal
Pan troglodytes	0.276102088	431	Primates	Mammal
Pan paniscus	0.276102088	431	Primates	Mammal
Gorilla gorilla	0.273781903	431	Primates	Mammal
Pongo abellii	0.299303944	431	Primates	Mammal
Pongo pygameus	0.299303944	431	Primates	Mammal
Hylobates lar	0.278422274	431	Primates	Mammal
Nomascus leucogenys	0.276744186	430	Primates	Mammal
Macaca mulatta	0.294663573	431	Primates	Mammal
Papio anubis	0.287703016	431	Primates	Mammal
Chlorocebus sabaeus	0.315545244	431	Primates	Mammal
Saimiri boliviensis boliviensis	0.341067285	431	Primates	Mammal
Callithrix jacchus	0.338747100	431	Primates	Mammal
Galeopterus variegatus	0.274418605	430	Dermoptera	Mammal
Tarsius syrichta	0.274418605	430	Primates	Mammal
Tupaia chinensis	0.362186788	439	Scandentia	Mammal
Mus musculus	0.258741259	429	Rodentia	Mammal
Cricetulus griseus	0.338785047	428	Rodentia	Mammal
Rattus norvegicus	0.254079254	429	Rodentia	Mammal
Octodon degus	0.266203704	432	Rodentia	Mammal
Oryctolagus cuniculus	0.335648148	432	Lagomorpha	Mammal
Ochotona princeps	0.315668203	434	Lagomorpha	Mammal
Physeter catodon	0.313225058	431	Cetacea	Mammal
Orcinus orca	0.313225058	431	Cetacea	Mammal
Camelus ferus	0.269141531	431	Artiodactyla	Mammal
Bos taurus	0.310904872	431	Artiodactyla	Mammal
Equus caballus	0.395348837	430	Perissodactyla	Mammal
Ailuropoda melanoleuca	0.394859813	428	Carnivora	Mammal
Felis catus	0.365740741	432	Carnivora	Mammal
Canis lupus familiaris	0.374418605	430	Carnivora	Mammal
Vicugna pacos	0.271461717	431	Artiodactyla	Mammal
Panthera tigris	0.390697674	430	Carnivora	Mammal
Mustela putorius furo	0.360277136	433	Carnivora	Mammal
Odobenus rosmarus divergens	0.321759259	432	Carnivora	Mammal
Leptonychotes weddellii	0.374133949	433	Carnivora	Mammal
Ceratotherium simum simum	0.329466357	431	Perissodactyla	Mammal
Eptesicus fuscus	0.264501160	431	Chiroptera	Mammal
Myotis brandtii	0.308584687	431	Chiroptera	Mammal
Pteropus alecto	0.271461717	431	Chiroptera	Mammal
Condylura cristata	0.287383178	428	Soricomorpha	Mammal
Chrysochloris asiatica	0.320185615	431	Afrosoricida	Mammal
Erinaceus europaeus	0.294252874	435	Erinaceomorpha	Mammal

Table S4.1. Disorder Proportion for FOXP3 orthologues (continued).

Species	Disorder Proportion	AA	Order	Class
Elephantulus edwardii	0.299303944	431	Macroscelidea	Mammal
Echinops telfairi	0.314814815	432	Afrosoricida	Mammal
Sorex araneus	0.331018519	432	Soricomorpha	Mammal
Orycteropus afer afer	0.354988399	431	Tubulidentata	Mammal
Loxodonta africana	0.309133489	427	Proboscidea	Mammal
Trichechus manatus latirostris	0.365967366	429	Sirenia	Mammal
Dasypus novemcinctus	0.338747100	431	Cingulata	Mammal
Ornithorhynchus anatinus	0.198598131	428	Monotremata	Mammal
Monodelphis domestica	0.166666667	462	Didelphimorphia	Mammal
