# Supplementary material for: FOXP in Tetrapoda: Intrinsically Disordered Regions, Short Linear Motifs and their evolutionary significance
Source: Genet Mol Biol. 2017 Mar 2;40(1):181–90. doi: 10.1590/1678-4685-GMB-2016-0115 (PMC5409772; doi:10.1590/1678-4685-GMB-2016-0115)
Supplement: Table S4.2 [file 1415-4757-gmb-1678-4685-GMB-2016-0115-Suppl07.pdf]

Table S4.2. Ordered regions for FOXP3 orthologues.

Species	Structured Region			
Homo sapiens	89-182	189-270		278-413
Pan troglodytes	89-184	189-270		278-413
Pan paniscus	89-184	189-270		278-413
Gorilla gorilla	89-184	189-270		278-413
Pongo abellii	89-259			281-413
Pongo pygameus	89-259			281-413
Hylobates lar	89-182	189-270		277-413
Nomascus leucogenys	89-184	189-269		276-412
Macaca mulatta	89-182	190-263		278-413
Papio anubis	89-182	190-263		278-413
Chlorocebus sabaeus	89-182	190-259	278-289	300-413
Saimiri boliviensis boliviensis	90-175	192-260	279-290	301-413
Callithrix jacchus	89-174	191-259	278-289	300-413
Galeopterus variegatus	88-108	123-183		188-413
Tarsius syrichta	89-173			190-412
Tupaia chinensis	98-115	138-266		287-421
Sorex araneus	94-108	136-183		190-415
Mus musculus	89-107	111-269		276-415
Rattus norvegicus	92-297			302-415
Cricetulus griseus	91-106	123-257	279-295	301-414
Octodon degus	91-289			300-417
Oryctolagus cuniculus	90-252			302-415
Ochotona princeps	93-122	127-175	193-297	305-417
Physeter catodon	89-182		190-258	278-413
Orcinus orca	89-182		190-258	278-413
Camelus ferus	90-264			277-414
Bos taurus	90-182	189-258		277-413
Ailuropoda melanoleuca	94-109	134-174	193-266	284-410
Felis catus	97-103	128-171	190-270	276-415
Canis lupus familiaris	91-104	131-171	190-270	277-411
Vicugna pacos	90-271			277-414
Panthera tigris	96-104	123-171	190-270	293-413
Mustela putorius furo	100-106	135-174		191-415
Odobenus rosmarus divergens	93-117	131-183		191-414
Leptonychotes weddellii	91-118	130-183	190-260	300-415
Equus caballus	89-106	125-171	191-263	292-413
Ceratotherium simum simum	92-171		192-265	277-414
Eptesicus fuscus	90-183			190-415
Myotis brandtii	93-107	124-184		189-415
Pteropus alecto	89-182			190-415
Condylura cristata	90-171			189-411
Chrysochloris asiatica	97-107	124-270		279-414

Table S4.2. Ordered regions for FOXP3 orthologues (continued).

Species	Structured Region			
Erinaceus europaeus	96-111	116-188	193-274	281-418
Elephantulus edwardii	20-53	127-265	277-290	300-415
Echinops telfairi	92-119	127-172	190-265	278-414
Orycteropus afer afer	97-104	126-174	189-271	277-414
Loxodonta africana	94-170		190-268	274-412
Trichechus manatus latirostris	123-175		190-257	276-412
Dasypus novemcinctus	92-104	133-182		189-413
Monodelphis domestica	24-44			85-450
