# Supplementary material for: FOXP in Tetrapoda: Intrinsically Disordered Regions, Short Linear Motifs and their evolutionary significance
Source: Genet Mol Biol. 2017 Mar 2;40(1):181–90. doi: 10.1590/1678-4685-GMB-2016-0115 (PMC5409772; doi:10.1590/1678-4685-GMB-2016-0115)
Supplement: Table S5.2 [file 1415-4757-gmb-1678-4685-GMB-2016-0115-Suppl09.pdf]

Table S5.2. Ordered regions for FOXP4.

Species	Structured Region						
Homo sapiens		66-71	219-228	308-355	391-396	452-542	558-581
Pan paniscus		66-71	219-228	308-355	391-396	452-542	558-581
Gorilla gorilla		66-71	219-228	308-355	391-396	452-542	558-581
Pongo abelli		66-71	219-228	308-355	391-396	452-542	558-581
Nomascus leucogenys		68-71	219-228	308-355	391-396	453-542	558-581
Macaca mulatta			219-228	308-355	391-396	452-542	558-581
Papio anubis			219-228	308-355	391-396	452-542	558-581
Chlorocebus sabaeus			219-228	308-355	391-396	452-542	558-581
Callithrix jacchus			219-228	308-355	388-396	451-541	550-582
Saimiri boliviensis			219-228	308-351	391-396	452-542	558-581
Otolemur garnettii			220-229	309-356	392-397	454-543	559-589
Galeopterus variegatus			220-229	309-354	387-398	454-543	559-588
Tupaia chinensis		67-74	223-230	310-357		438-527	543-566
Mus musculus			224-230	314-361	396-402	458-547	563-585
Rattus norvegicus			223-225	313-360	395-401	456-546	562-582
Cricetulus griseus			224-234	314-361	396-402	458-547	563-583
Octodon degus				312-358	384-400	454-544	560-583
Cavia porcellus				319-363	391-407	461-551	567-589
Ochotona princeps			225-232	309-359	396-400	458-547	563-589
Orcinus orca			224-236	315-362	398-404	459-549	656-586
Physeter catodon			223-232	310-359	395-400	456-546	562-584
Bos taurus			224-233	313-358		458-547	563-586
Vicugna pacos			226-235	315-362	398-403	460-549	565-587
Camelus ferus			226-235	315-362	398-403	460-549	565-587
Sus scrofa			226-235	315-363	388-401	460-549	565-595
Canis lupus			220-229	309-356	392-397	454-543	559-581
Mustela putorius furo			219-228	308-355	391-395	453-542	558-580
Ailuropoda melanoleuca	21-37	85-94		273-321	357-362	422-508	524-547
Odobenus rosmarus			219-228	308-355	391-397	453-542	558-588
Leptonychotes weddelli			221-230	310-357	387-398	454-543	559-589
Eptesicus fuscus			220-229	309-356	392-395	454-543	559-581
Myotis brandtii			220-229	309-356	392-395	454-543	559-580
Pteropus alecto			222-227	311-358	394-399	456-545	561-583
Equus caballus			224-231	311-358	393-397	459-545	561-583
Ceratotherium simum			220-229	303-352	391-398	454-543	559-582
Chrysochloris asiatica			225-233	314-356	397-400	463-548	564-590
Condylura cristata			222-231	308-360	382-410	455-545	561-583
Elephantulus edwardii			222-229	309-352	395-398	457-544	560-586
Erinaceus europaeus			234-243	317-368		477-566	582-605
Sorex araneus			228-237	314-360		466-555	571-594
Orycteropus afer afer			225-232	312-359	395-400	462-547	563-589
Loxodonta africana		71-78		314-361	397-401	459-548	564-586

Table S5.2. Ordered regions for FOXP4 (continued).

Species	Structured Region					
Trichechus manatus latirostris		226-234	313-360	396-400	462-548	564-587
Taeniopygia guttata	63-66	212-233	302-347		437-532	548-579
Serinus canaria	64-71	214-236	305-350		437-535	551-583
Pseudopodoces humilis	64-71	213-235	303-349		435-534	550-582
Falco peregriunus		213-235	304-349		435-534	550-582
Calypte anna		196-221	287-332		418-517	533-565
Aptenodytes forsteri	51-58	201-223	292-337		423-522	538-570
Gallus gallus		207-229	298-343		430-528	544-576
Anas platyrhynchos	56-59	208-230	299-344			430-569
Alligator mississipiensis		213-235	299-349		439-534	550-582
Alligator sinensis		209-231	295-345		435-530	546-578
Python bivittatus	62-69	212-234	298-348		434-533	549-580
Anolis carolinensis	64-70	214-249	297-350	376-385	436-535	551-582
Chrysemys picta bellii		211-233	301-348		438-535	548-580
Chelonia mydas		210-232	300-347		437-534	547-580
Pelodiscus sinensis		207-229	297-344		434-531	544-586
Xenopus tropicalis			280-331		411-517	530-558
Xenopus laevis			276-327		406-513	526-544
