# Supplementary material for: FOXP in Tetrapoda: Intrinsically Disordered Regions, Short Linear Motifs and their evolutionary significance
Source: Genet Mol Biol. 2017 Mar 2;40(1):181–90. doi: 10.1590/1678-4685-GMB-2016-0115 (PMC5409772; doi:10.1590/1678-4685-GMB-2016-0115)
Supplement: Table S6.2 [file 1415-4757-gmb-1678-4685-GMB-2016-0115-Suppl11.pdf]

Table S6.2. Whole protein comparison for FOXP2 linear motifs content.

	Linear Motifs	CLV_C14_Caspase3-7	DEG_APCC_DBOX_1	DEG_SIAH_1	DOC_CKS1_1	DOC_CYCLIN_1	DOC_MAPK_1	DOC_PP2B_2	DOC_USP7_1	DOC_WW_Pin1_4	LIG_14-3-3_3	LIG_CtBP_PxDLS_1	LIG_FHA_1	LIG_FHA_2	LIG_NRBOX	LIG_PCNA_PIPBox_1	LIG_PTAP_UEV_1	LIG_SUMO_SBM_1	LIG_SUMO_SBM_2	MOD_CK1_1	MOD_CK2_1	MOD_GSK3_1	MOD_NEK2_1	MOD_NEK2_2	MOD_PIKK_1	MOD_PKA_1	MOD_PKA_2	MOD_PLK	MOD_ProDKin_1	MOD_SUMO	TRG_NES_CRM1_1	Total
**Mammals**	*Homo sapiens*	2	1	1	6	4	1	1	4	16	1	2	5	2	1		1	2	6	16	7	33	7	1	3	3	6	1	16	1		150
	Pan troglodytes	2	1	1	6	4	1	1	4	16	1	2	5	2	1		1	2	6	15	7	33	7	1	3	3	6	1	16	1		149
	Gorilla gorilla	2	1	1	6	4	1	1	4	16	1	2	5	2	1		1	2	6	15	7	33	7	1	3	3	6	1	16	1		149
	Pongo abelii	2	1	1	6	4	1	1	3	16	1	2	5	2	1		1	2	6	15	7	33	7	1	3	3	6	1	16	1		148
	Nomascus leucogenys	2	1	1	6	4	1	1	4	16	1	2	5	2	1		1	2	6	15	7	33	7	1	3	3	6	1	16	1		149
	Macaca mulatta	2	1	1	6	4	1	1	4	16	1	2	5	2	1		1	2	6	15	7	33	7	1	3	3	6	1	16	1		149
	Papio anubis	2	1	1	6	4	1	1	4	16	1	2	5	2	1		1	2	6	15	7	33	7	1	3	3	6	1	16	1		149
	Chlorocebus sabaeus	2	1	1	6	4	1	1	4	16	1	2	5	2	1		1	2	6	15	7	33	7	1	3	3	6	1	16	1		149
	Saimiri boliviensis	2	1	1	6	4	1	1	4	16	1	2	5	2	1		1	2	6	15	7	33	7	1	3	3	6	1	16	1		149
	Callithrix jacchus	2	1	1	6	4	1	1	4	16	1	2	5	2	1		1	2	6	15	7	33	7	1	3	3	6	1	16	1		149
	Galeopterus variegatus	3	1	1	6	3			4	16	1	2	6	2	1		1	2	4	15	7	33	7	1	3	3	6	1	16	1		146
	Tupaia chinensis	2	1	1	6	4	1	1	4	16	1	2	5	2	1		1	2	6	15	7	33	7	1	3	3	6	1	16	1		149
	Mus musculus	2	1	1	6	4	1	1	4	16	1	2	5	2	1		1	2	6	15	7	33	7	1	3	3	6	1	16	1		149
	Rattus norvegicus	2		1	6	4	1	1	4	16	1	2	5	2	1		1	2	6	15	7	33	7	1	4	3	6	1	16	1		149
	Cricetulus griseus	2	1	1	6	4	1	1	4	16	1	2	5	2	1		1	2	6	15	7	32	7	1	3	3	6	1	16	1		148
	Octodon degus	2	1	1	6	4	1	1	4	16		2	5	2	1		1	2	6	16	7	32	7	1	3	3	6	1	16	1		148
	Oryctolagus cuniculus	2	1	1	6	4	1	1	4	16	1	2	5	2	1		1	2	6	15	7	33	7	1	3	3	6	1	16	1		149
	Ochotona princeps	2	1		6	4	2	1	5	16	1	2	5	2	1	1	1	2	6	14	7	32	7	1	3	3	6	2	16	1		150
	Physeter catodon	2	1	1	6	4	1	1	5	17	1	2	5	2	1		1	2	6	14	7	30	7	1	3	3	6	1	17	1		148
	Camelus ferus	2	1	1	6	4	1	1	4	16	1	2	5	2	1		1	2	6	15	7	33	7	1	4	3	6	1	16	1		150
	Vicugna pacos	2	1	1	6	4	1	1	4	16	1	2	5	2	1		1	2	6	15	7	33	7	1	4	3	6	1	16	1		150
	Ceratotherium simum simum	2	1	1	6	4	1	1	4	16	1	2	5	2	1		1	2	6	14	7	31	7	1	3	3	6	1	16	1		146

Table S6.2. Whole protein comparison for FOXP2 linear motifs content (continued).

	**Linear Motifs**	CLV_C14_Caspase3-7	DEG_APCC_DBOX_1	DEG_SIAH_1	DOC_CKS1_1	DOC_CYCLIN_1	DOC_MAPK_1	DOC_PP2B_2	DOC_USP7_1	DOC_WW_Pin1_4	LIG_14-3-3_3	LIG_CtBP_PxDLS_1	LIG_FHA_1	LIG_FHA_2	LIG_NRBOX	LIG_PCNA_PIPBox_1	LIG_PTAP_UEV_1	LIG_SUMO_SBM_1	LIG_SUMO_SBM_2	MOD_CK1_1	MOD_CK2_1	MOD_GSK3_1	MOD_NEK2_1	MOD_NEK2_2	MOD_PIKK_1	MOD_PKA_1	MOD_PKA_2	MOD_PLK	MOD_ProDKin_1	MOD_SUMO	TRG_NES_CRM1_1	Total
**Mammals**	*Odobenus rosmarus divergens*	2	1	1	6	4	1	1	4	16	1	2	5	2	1		1	2	6	16	7	34	7	1	3	3	6	1	16	1		151
	Panthera tigris	2	1	1	6	4	1	1	4	16	1	2	5	2	1		1	2	6	16	7	34	7	1	3	3	6	1	16	1		151
	Felis catus	2	1	1	6	4	1	1	4	16	1	2	5	2	1		1	2	6	16	7	34	7	1	3	3	6	1	16	1		151
	Myotis brandtii	3	1	1	6	4	1	1	5	17	1	2	5	3	1		1	3	6	13	7	34	7	1	3	3	6	1	17	1		154
	Eptesicus fuscus	3	1	1	6	4	1	1	5	17	1	2	5	3	1		1	3	6	13	7	34	7	1	3	3	6	1	17	1		154
	Pteropus alecto	2	1	1	6	4	1	1	4	16	1	2	6	2	1		1	2	6	15	7	33	7	1	3	3	6	1	16	1		150
	Erinaceus europaeus	3	1	1	6	4	1	1	4	16	1	2	6	2	1		1	2	6	14	7	35	7	1	3	3	6	1	16	1		152
	Condylura cristata	2	1	1	6	4	1	1	4	16	1	2	5	2	1		1	2	6	15	7	33	7	1	3	3	6	1	16	1		149
	Echinops telfairi	1	1	1	6	4	1	1	5	16	1	2	5	2	1		1	2	6	15	7	31	7	1	3	3	6	1	16	1		147
	Chrysochloris asiatica	2	1	1	6	4	1	1	4	16	1	2	5	2	1		1	2	6	16	7	33	7	1	3	3	6	1	16	1		150
	Elephantulus edwardii	2	1	1	6	4	1	1	3	16	1	2	5	2	1		1	2	6	15	7	32	7	1	3	3	6	1	16	1		147
	Orycteropus afer afer	3	1	1	6	4	1	1	4	16	1	2	5	1	1		1	2	6	15	7	33	7	1	3	3	6	1	16	1		149
	Trichechus manatus latirostris	2	1	1	6	4	1	1	4	16	1	2	5	2	1		1	2	6	15	7	33	7	1	3	3	6	1	16	1		149
	Loxodonta africana	2	1	1	6	4	1	1	4	16	1	2	5	2	1		1	2	6	15	7	33	7	1	3	3	6	1	16	1		149
	Monodelphis domestica	2	1	1	6	4	1	1	5	17		2	4	2	1		1	2	4	13	7	32	7	1	3	3	6	1	17	1		145
**Birds**	*Serinus canaria*	2	1	1	6	4	1	1	5	16		2	5	2	1		1	2	6	13	7	33	7	1	3	3	6	1	16	1		147
	Zonotrichia albicollis	2	1	1	6	4	1	1	5	16		2	5	2	1		1	2	6	13	7	33	7	1	3	3	6	1	16	1		147
	Ficedula albicollis	2	1	1	6	4	1	1	5	16		2	5	2	1		1	2	6	13	6	33	7	1	3	3	6	1	16	1		146
	Taeniopygia guttata	2	1	1	6	4	1	1	5	16		2	5	2	1		1	2	6	14	7	33	7	1	3	3	6	1	16	1		148
	Manacus vitellinus	2	1	1	6	4	1	1	5	16		2	5	2	1		1	3	6	13	7	32	6	1	3	3	6	1	16	1	2	148
	Melopsittacus undulatus	2	1	1	6	4	1	1	5	16		2	5	2	1		1	2	6	13	7	33	7	1	3	3	6	1	16	1		147
	Falco peregrinus	2	1	1	6	4	1	1	5	16		2	5	2	1		1	2	6	13	7	33	7	1	3	3	6	1	16	1	1	148

Table S6.2. Whole protein comparison for FOXP2 linear motifs content (continued).

	Linear Motifs	CLV_C14_Caspase3-7	DEG_APCC_DBOX_1	DEG_SIAH_1	DOC_CKS1_1	DOC_CYCLIN_1	DOC_MAPK_1	DOC_PP2B_2	DOC_USP7_1	DOC_WW_Pin1_4	LIG_14-3-3_3	LIG_CtBP_PxDLS_1	LIG_FHA_1	LIG_FHA_2	LIG_NRBOX	LIG_PCNA_PIPBox_1	LIG_PTAP_UEV_1	LIG_SUMO_SBM_1	LIG_SUMO_SBM_2	MOD_CK1_1	MOD_CK2_1	MOD_GSK3_1	MOD_NEK2_1	MOD_NEK2_2	MOD_PIKK_1	MOD_PKA_1	MOD_PKA_2	MOD_PLK	MOD_ProDKin_1	MOD_SUMO	TRG_NES_CRM1_1	Total
**Birds**	*Aptenodytes forsteri*	2	1	1	6	4	1	1	5	16		2	5	2	1		1	2	6	13	7	33	7	1	3	3	6	1	16	1		147
	Calypte anna	2	2	1	6	4	1	1	5	16		2	5	2	1		1	3	6	13	7	33	7	1	3	3	6	1	16	1	1	150
	Anas platyrhynchos	2	1	1	6	4	1	1	4	16		2	5	2	1		1	2	6	13	7	33	7	1	3	3	6	1	16	1		146
	Gallus gallus	2	1	1	6	4	1	1	4	16		2	5	2	1		1	2	6	13	7	33	7	1	3	3	6	1	16	1		146
**Reptilia**	*Python bivittatus*	2	1	1	6	4	1	1	4	16		2	5	3	1		1	1	6	13	7	30	6	1	3	3	6	1	16	1		142
	Anolis carolinensis	3	1	1	6	5	1	1	5	16		2	5	2	1		1	2	6	15	7	31	8	1	3	3	6	1	16	1		150
	Chelonia mydas	2	2	1	6	4	1	1	6	16		2	5	2	1		1	2	6	13	7	33	7	1	3	3	6	1	16	1	1	150
	Pelodiscus sinensis	2	1	1	6	4	1	1	6	16		2	5	2	1		1	2	6	13	7	32	6	1	2	3	6	1	16	1		145
**Amphibian**	*Xenopus laevis*	2	1	1	6	4	1	1	4	15	1	3	8	2	1		1	2	6	15	7	32	8	1	3	3	6	1	15	1		151
